# *Nedd4-2* ablation in kidney improves glycaemic control in diabetic mice

**DOI:** 10.1038/s41419-025-07826-3

**Published:** 2025-07-05

**Authors:** Jantina A. Manning, Shilpanjali Jesudason, Paul A. B. Moretti, Stuart M. Pitson, Angela S. Y. Chou, Meriam Shabbar, Sonia Saad, Carol Pollock, Sharad Kumar

**Affiliations:** 1 Centre for Cancer Biology, University of South Australia and SA Pathology, Adelaide, SA Australia; 2 Adelaide University, Adelaide, SA Australia; 3 Central Northern Adelaide Renal and Transplantation Service (CNARTS), Royal Adelaide Hospital, Adelaide, SA Australia; 4 Faculty of Health and Medical Sciences, The University of Adelaide, Adelaide, SA Australia; 5 NSW Health Pathology, Royal North Shore Hospital, The University of Sydney, Sydney, NSW Australia; 6 Kolling Institute of Medical Research, Royal North Shore Hospital and The University of Sydney, Sydney, NSW Australia

**Keywords:** Molecular biology, Cell signalling

## Abstract

NEDD4-2, a ubiquitin ligase, regulates a number of ion channels and transporters by promoting their ubiquitination, internalisation and degradation, thereby affecting many signalling and physiological outcomes. Loss of this gene in mice results in tubular cell death and a chronic kidney disease (CKD)-like phenotype due to aberrant Na^+^ transport, caused by elevated expression of NEDD4-2 substrates including the epithelial sodium channel (ENaC). One of the biggest risk factors for CKD is diabetes, as up to 50% of diabetic patients develop diabetic kidney disease (DKD). Reduced levels of *Nedd4-2* are associated with DKD in patients, therefore we investigated if this gene contributes to the development of this disease. In a diabetic (db/db) mouse model that develops DKD, we observed reduced expression of *Nedd4-2* that correlated with disease progression. Substrates of NEDD4-2, including ENaC, were elevated in db/db mice, suggesting that NEDD4-2 dysfunction is involved in disease pathology. Intriguingly, genetic ablation of *Nedd4-2* in this diabetic model did not exacerbate kidney disease severity beyond *Nedd4-2* loss alone, but corrected metabolic parameters via a reduction of aldosterone levels, restoration of insulin signaling and reduced blood glucose levels. Hence, we conclude that a reduced *Nedd4-2* level is detrimental for kidney health, however unexpectedly improves glycemic control in diabetes.

## Introduction

Diabetic kidney disease (DKD) occurs in up to 50% of people with diabetes and is the leading cause of chronic kidney disease (CKD), a major contributor to end-stage kidney disease (ESKD) [1]. DKD can develop from type 1 diabetes mellitus (T1DM, absolute insulin deficiency) or type 2 diabetes mellitus (T2DM, relative insulin deficiency and resistance) [2]. This disease is characterised by raised urinary albumin or changes in glomerular filtration rate (GFR), stemming from long-standing hyperglycaemia and a combination of metabolic changes that cause chronic pathological hyperfiltration, inflammation, fibrosis, renal cell loss, damage and death [3]. Development of DKD in patients depends on modifiable factors such as the extent of hypertension and normalisation of the metabolic milieu, as well as genetic components [4], resulting in altered gene expression and compromised cellular and tissue integrity [3].

Linkage analysis of multiple populations has revealed a susceptibility locus of DKD encompassing the region where *NEDD4L* (human homologue of *Nedd4-2*) resides [5] and polymorphisms within *NEDD4L* have been associated with T2DM and DKD [6–8]. Furthermore, a study utilising unbiased single-nucleus RNA sequencing of human kidneys from patients with diabetes showed a significant down-regulation of *NEDD4L* in DKD, correlating with aberrant electrolyte transport [9]. Together, these observations suggest a pathophysiological role for *NEDD4L* in DKD.

NEDD4L is a member of the NEDD4 family of ubiquitin ligases that transfer ubiquitin onto substrate proteins, targeting them for turnover, recycling or specific signalling platforms, thus regulating multiple physiological processes and signalling outcomes [10]. NEDD4L has been shown to primarily regulate membrane channels and transporters such as the epithelial sodium channel (ENaC), Na^+^Cl^−^ cotransporter (NCC) and the sodium glucose cotransporter 1 (SGLT1) [10]. These channels are implicated in DKD, as the dysregulation of glucose and ion transport contributes to renal damage and disease progression [11]. Our previous studies have established that kidney-specific deletion of *Nedd4-2* causes progressive kidney disease with some features akin to CKD, characterised by tubular cell death, dilated tubules, interstitial fibrosis, polydipsia, polyuria and hypertension [12, 13]. Similar to human DKD, elevated ENaC and NCC expression are observed in these mice [10], with increased serum Na^+^, decreased serum K^+^, and increased urinary K^+^ excretion [12]. High dietary Na^+^ exacerbates kidney pathology, leading to progression to ESKD [14].

Lepr^*db/db*^ (db/db) mice provide a commonly used model of T2DM due to mutation in the *leptin receptor* gene, with susceptibility to obesity and insulin resistance. By 3–4 months of age, db/db mice display pathological features of human DKD and a decline in kidney function [15]. In this study, we examined the kidney-specific expression and function of NEDD4-2 in the db/db mouse model of T2DM and found a reduction of this gene correlated with disease progression. Further genetic reduction of *Nedd4-2*, specifically in the kidneys of db/db mice, did not exacerbate nephropathy beyond *Nedd4-2* loss alone. However, surprisingly, *Nedd4-2* deficiency in diabetic kidneys corrected metabolic parameters via reduction of elevated aldosterone, improved insulin signalling and restored blood glucose levels. Together, these results suggest that despite *Nedd4-2* deficiency resulting in kidney injury, paradoxically, it is protective against elevated blood glucose in diabetes.

## Results

### Nedd4-2 expression is reduced in db/db kidneys

**Fig. 1:**
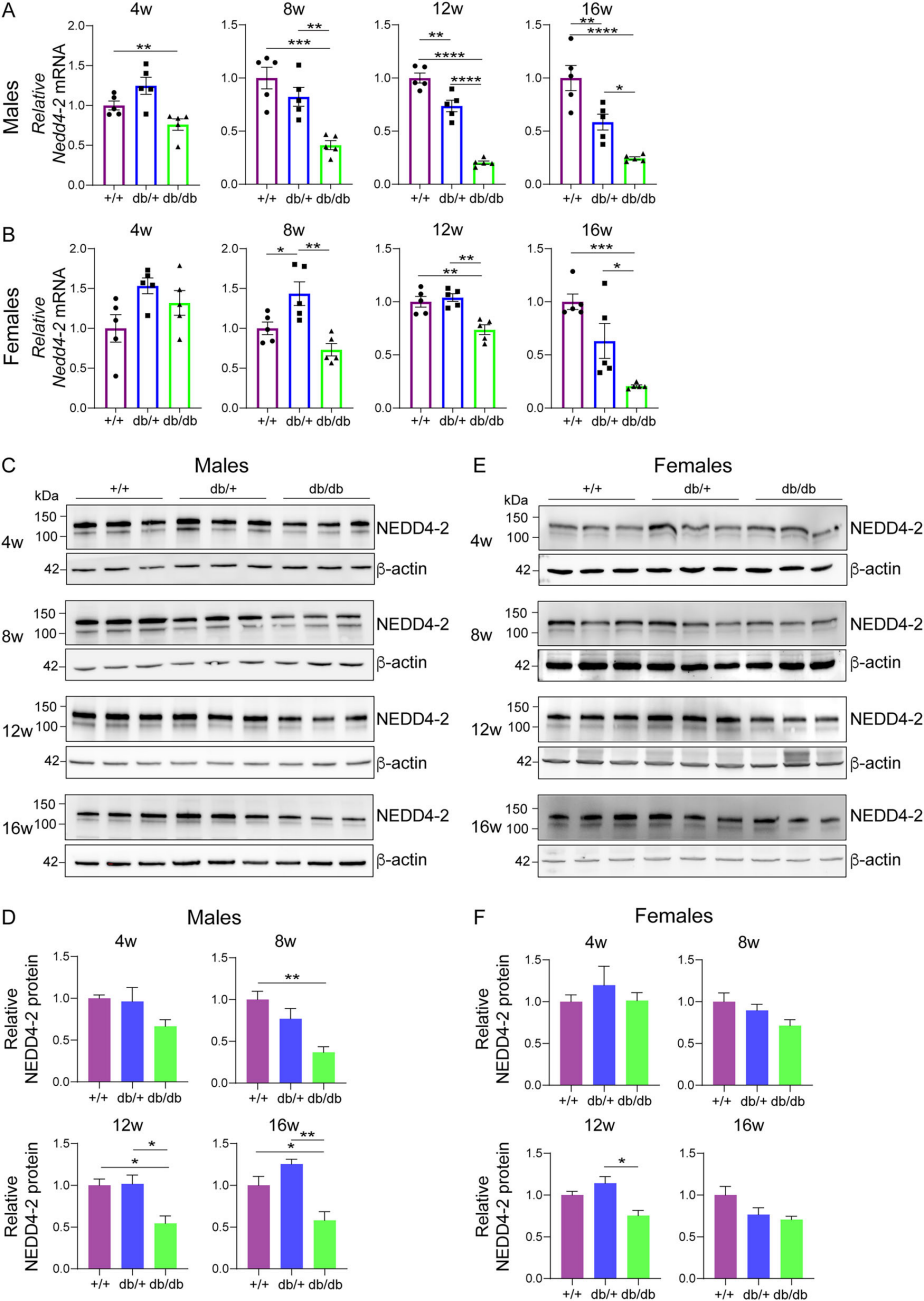
*Nedd4-2* expression is reduced in db/db kidneys. qPCR for *Nedd4-2* in kidneys of n = 5 individual mice at 4, 8, 12 and 16 weeks (w) normalised to *TBP* housekeeping gene: males (**A**) or females (**B**). **C** Male whole kidney lysates (n = 3 each genotype) assessed for NEDD4-2 and quantitated relative to β-actin in (**D**). **E** Female whole kidney lysates (n = 3 each genotype) assessed for NEDD4-2 and quantitated relative to β-actin in (**F**). Data presented as mean ± SEM for qPCR and mean ± SD for immunoblots, analysed by one-way ANOVA. *P < 0.05, **P < 0.01, ***P < 0.005, ****P < 0.001.

**Fig. 2:**
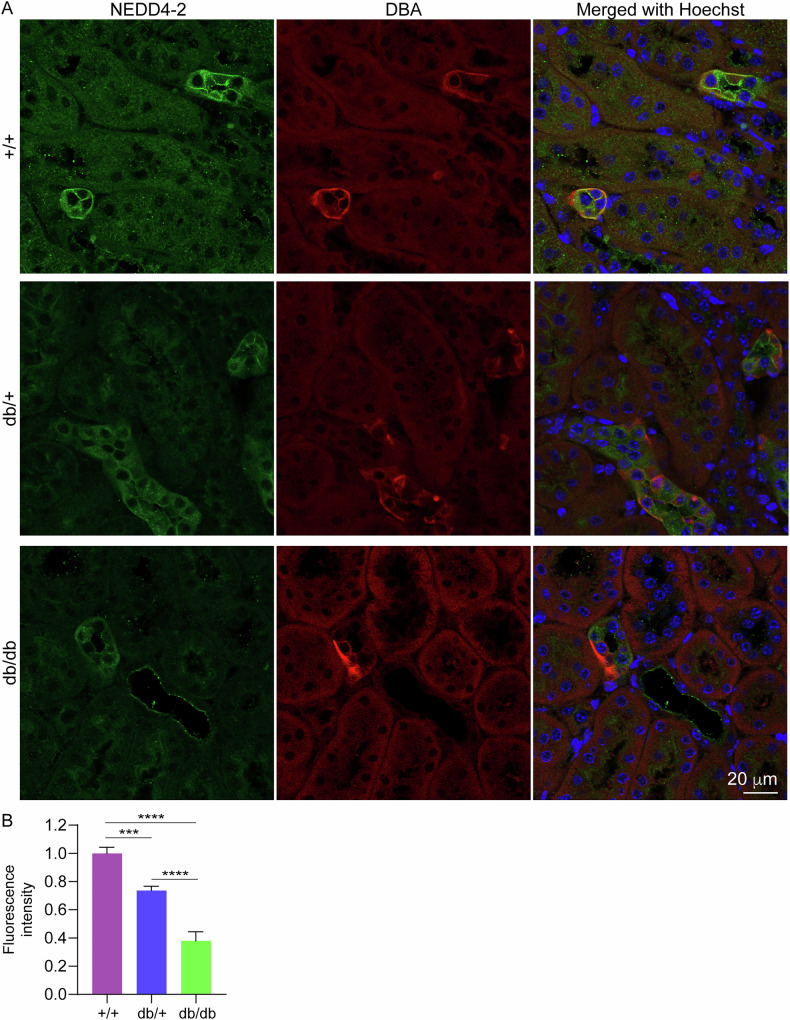
NEDD4-2 staining is reduced in db/db kidneys. **A** Immunofluorescence of NEDD4-2 with collecting duct marker DBA and Hoechst in 12-week-old male kidneys. **B** Fluorescence intensity per tubule area quantitated in (**B**) from n = 3 mice (10 tubules each). Data presented as mean ± SEM and analysed by one-way ANOVA. ***P < 0.005, ****P < 0.001.

Male and female db/db mice (B6.BKS(D)-*Lepr*^*db*^/J) develop hyperglycaemia from 6 weeks of age, albuminuria from 8 weeks and renal pathology by 12–16 weeks [15]. To determine the expression of *Nedd4-2* during the progression of DKD, mRNA levels were quantitated in kidneys from 4 weeks of age. In male mice, there was a significant reduction in *Nedd4-2* mRNA in db/db mice at 4 weeks (Fig. 1A), before hyperglycaemia and obesity have developed (Supplementary Figs. 1, 2). This reduction increased with age, until the end point of the study at 16 weeks. Heterozygous male mice (db/+) displayed a smaller decrease in *Nedd4-2* levels from 12 weeks. Female mice followed a similar trend, but *Nedd4-2* was not reduced in db/+, rather there was a small increase at 8 weeks (Fig. 1B). Compared to +/+ (wild-type, WT), a significant reduction in *Nedd4-2* was observed at 12 and 16 weeks in females (Fig. 1B). At the protein level in whole kidney lysates, NEDD4-2 was significantly reduced from 8 weeks of age in db/db male mice (Fig. 1C, D), to a lesser extent not reaching significance in females (Fig. 1E, F). NEDD4-2 levels in heterozygous mice were similar to WT. Staining of WT kidneys from 12-week-old male mice revealed strong expression of NEDD4-2 in distal tubules or collecting ducts, demonstrated by co-localisation with the collecting duct marker dolichos biflorus agglutinin (DBA) (Fig. 2A). A moderate reduction of NEDD4-2 was observed in db/+ kidneys, and a greater reduction in db/db kidneys (Fig. 2A, B).

### NEDD4-2 substrates are elevated in db/db kidneys

**Fig. 3:**
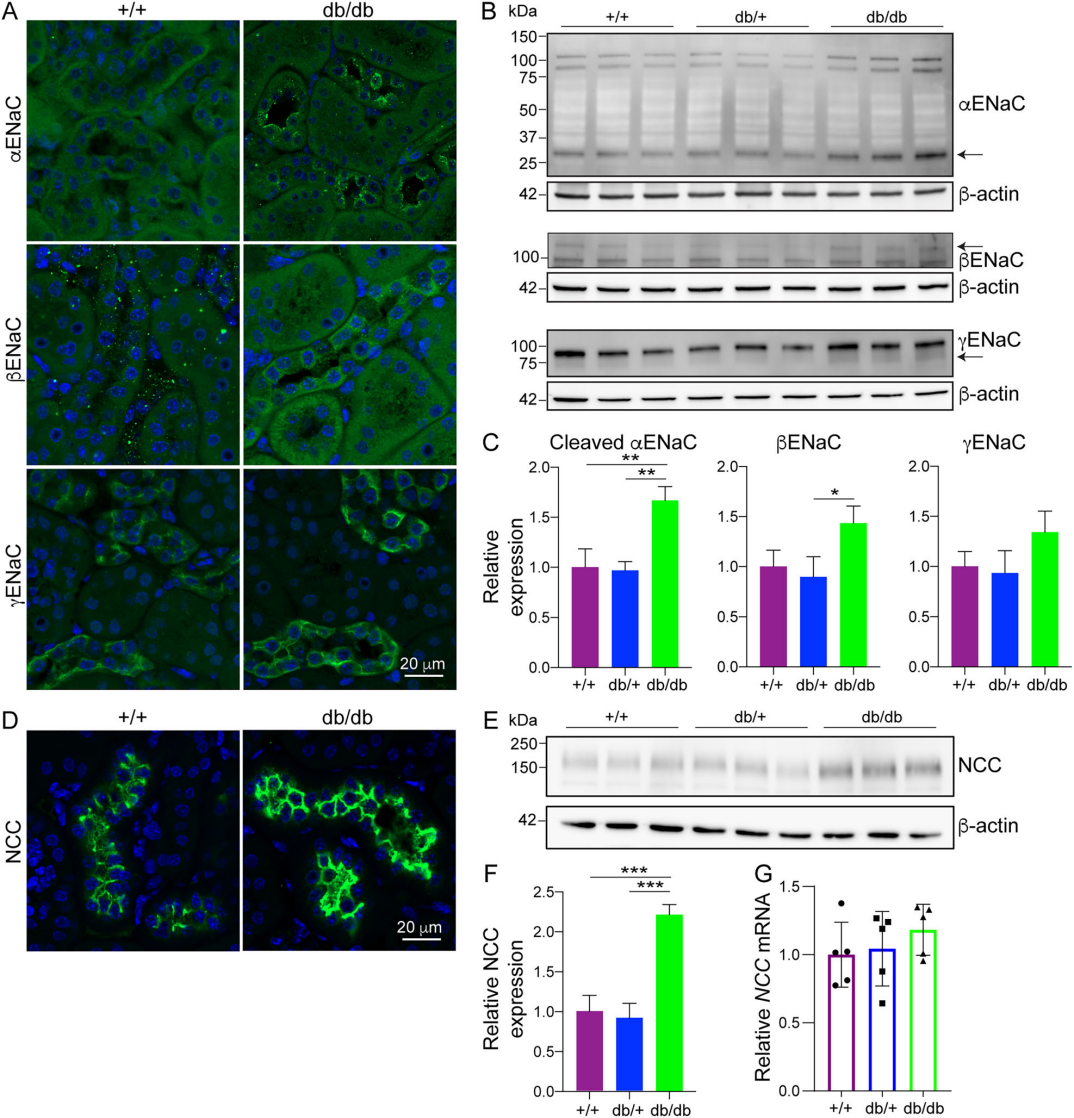
NEDD4-2 substrates are elevated in db/db kidneys. **A** Immunofluorescence of 12-week-old male kidneys. **B** Male whole kidney lysates, ENaC band indicated by arrow quantitated relative to β-actin in (**C**), n = 3 mice. **D** Immunofluorescence of 12-week-old male kidneys. **E** Male whole kidney lysates, NCC quantitated relative to β-actin in (**F**), n = 3. **G** qPCR for NCC in 12-week-old kidneys, n = 5. Data presented as mean ± SD for immunoblots (**C**, **F**) or mean ± SEM for qPCR (**G**) and analysed by one-way ANOVA. *P < 0.05, **P < 0.01, ****P < 0.001.

To assess if the activity of NEDD4-2 was also reduced in db/db kidneys, levels of known NEDD4-2 substrates were investigated. Immunocytochemistry of α, β and γENaC subunits revealed increased intensity in db/db kidneys compared to WT (Fig. 3A). All three ENaC subunits are processed to form the active membrane associated ENaC, which is regulated by NEDD4-2 [12, 16]. At the total protein level, whilst immature ENaC subunits were similar in all groups (Supplementary Fig. 3A), the active cleaved form of αENaC was significantly increased in db/db mice, as well as the mature glycosylated form of βENaC, with a trend towards increased mature cleaved γENaC (Fig. 3B, C). Expression of another NEDD4-2 substrate, NCC, was also increased in kidney tubules of db/db mice (Fig. 3D), as was the total protein level (Fig. 3E). Importantly, the mRNA level of *NCC* was not different between WT and db/db mice (Fig. 3G), suggesting that post-translational control, likely by NEDD4-2-mediated ubiquitination and degradation, is responsible for the increase in NCC protein in db/db mice.

### Genetic reduction of Nedd4-2 in db/db mice leads to increased substrate expression

**Fig. 4:**
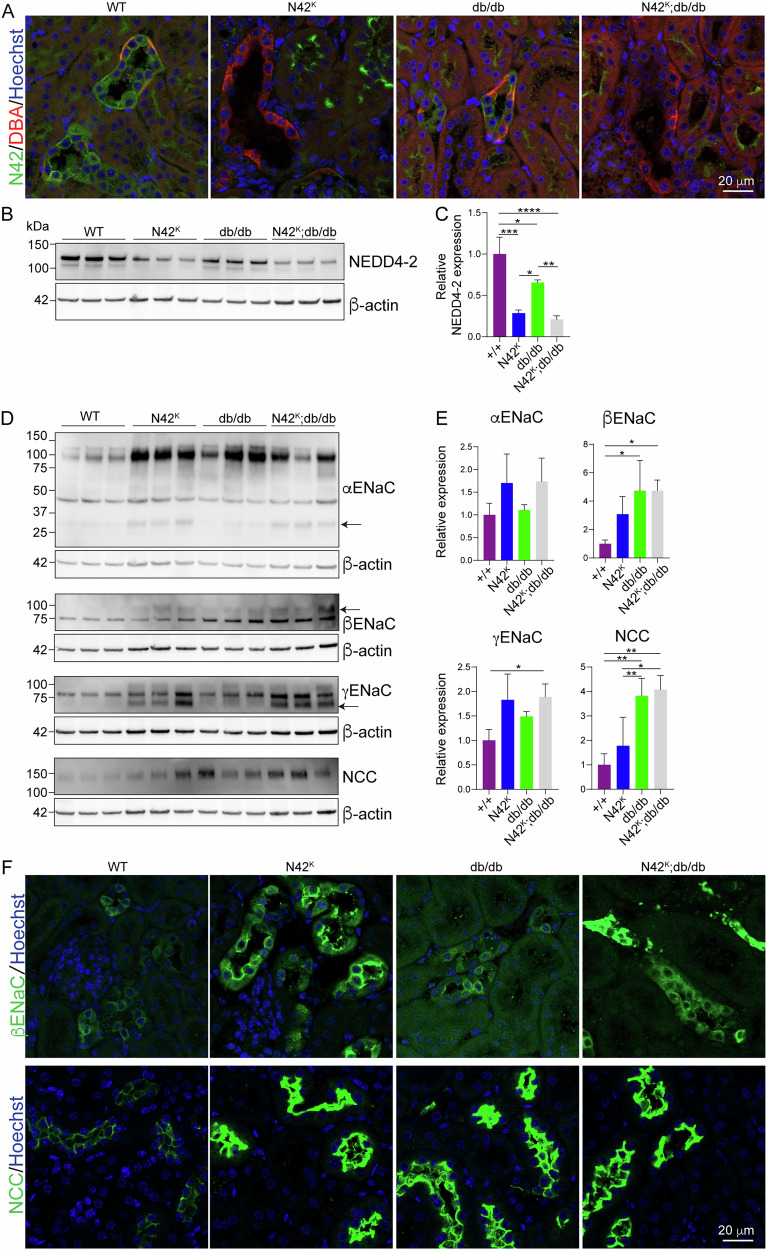
Genetic reduction of *Nedd4-2* in db/db mice leads to increased substrate expression. **A** Immunofluorescence of 12-week-old male kidneys. **B** Male whole kidney lysates assessed for NEDD4-2 and quantitated relative to β-actin in (**C**), n = 3. **D** Kidney lysates assessed for ENaC and NCC, band indicated by arrow quantitated relative to β-actin in (**E**), n = 3. **F** Immunofluorescence for βENaC or NCC in kidneys. Data presented as mean ± SD and analysed by one-way ANOVA. *P < 0.05, **P < 0.01, ****P < 0.001.

To investigate whether the reduced renal NEDD4-2 in the db/db model was a compensatory mechanism in the development of DKD or was contributing to disease severity, we exacerbated this by completely ablating NEDD4-2 from the renal tubules of db/db mice. To this end, kidney tubule-specific *Nedd4-2* deficient mice (N42^K^) were crossed into the B6.BKS(D)-*Lepr*^*db*^/J line to generate diabetic N42^K^;db/db mice. Previous analysis of the N42^K^ mice has revealed the onset of kidney damage and disease by 20 days of age, with tubular dilation and damage, mesenchymal infiltration and fibrosis [12, 14]. WT mice (for both *Nedd4-2* and *Lepr* B6.BKS, i.e normal NEDD4-2 levels and non-diabetic) from this cross again displayed strong expression of NEDD4-2 within collecting ducts (Fig. 4A). NEDD4-2 expression was lost from the collecting ducts of N42^K^ mice; however, some expression remained in other parts of the nephron. As shown previously, db/db mice had reduced levels of NEDD4-2, which were further lost in the N42^K^;db/db mice. At the total protein level, reduced NEDD4-2 expression was observed in all three modified lines, with partial reduction in db/db kidneys and a stronger reduction in N42^K^ and N42^K^;db/db kidneys (Fig. 4B, C). The remaining NEDD4-2 expression in these kidneys is due to the tubule-specific knockout of NEDD4-2 in this line. Similar to previous results, there was increased expression of mature ENaC subunits and NCC, most striking in the N42^K^;db/db line (Fig. 4D, E) with an increase in immature α and βENaC in the db/db mice only (Supplementary Fig. 3B). Similarly, increased tubular staining of βENaC (as a representative ENaC subunit) and NCC were apparent in the N42^K^, db/db and N42^K^;db/db kidneys (Fig. 4F). These data show that lower NEDD4-2 levels correlate with increased substrate expression in N42^K^;db/db kidneys.

### Kidney pathology in N42^K^;db/db mice resembles NEDD4-2 loss

**Fig. 5:**
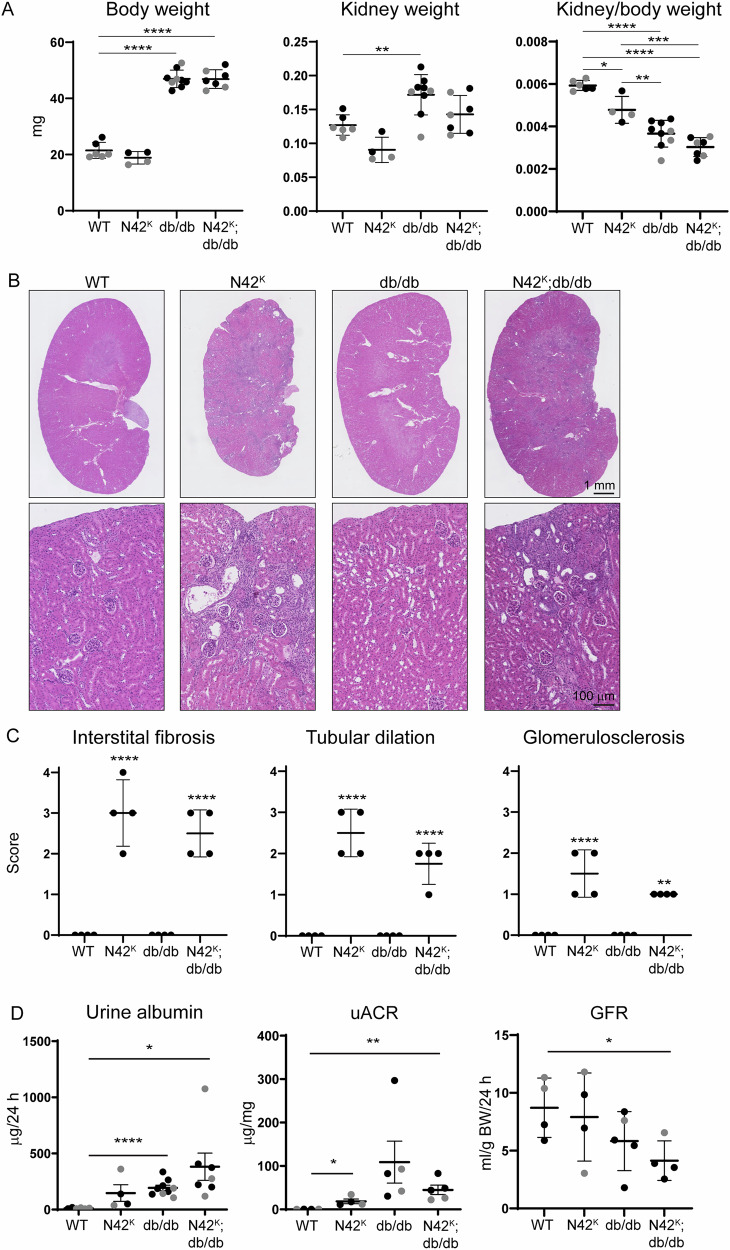
Kidney pathology and function in N42^K^;db/db mice. **A** Body weights of males (black dots) or females (grey dots), average of left and right kidney weights, or kidney/body weight ratios, n = 4-7. **B** Hematoxylin and Eosin staining of male kidneys at low or high magnification. **C** Kidneys were scored blind (range 0–4) for indicators of damage, n = 4 males. **D** 24 h urine samples were measured for albumin, or normalised to creatinine (uACR), and GFR calculated, n = 4–7. Data presented as mean ± SD and analysed by One-way ANOVA. **P < 0.01, ****P < 0.001. Significance in (**C**) was compared to WT.

Both the db/db mice and N42^K^;db mice were significantly heavier than WT and N42^K^ animals (Fig. 5A) (males and females). When compared to WT, kidney weights were elevated in db/db mice, but this was not significant in the N42^K^;db/db mice, and there was no difference in kidney/body weight ratios between db/db and N42^K^;db/db mice. As we have previously described, kidney pathology was evident in N42^K^ mice at 12 weeks of age, defined by dilated tubules, luminal debris and interstitial damage (Fig. 5B). At this age, the kidneys of db/db mice appeared normal, with no evidence of damage. Pathology of the N42^K^;db/db kidneys were similar to N42^K^ alone. Upon scoring various damage parameters, interstitial fibrosis, tubular dilation and glomerulosclerosis were significantly increased in N42^K^ and N42^K^;db/db kidneys when compared to WT (Fig. 5C). Casts were also present within tubular lumens in N42^K^ and N42^K^;db/db kidneys only (Fig. 5C). However, no difference was observed between N42^K^ and N42^K^;db/db kidneys. Elevated expression of markers of kidney damage TGFβ1, collagen 1, vimentin, IL1-β and KIM-1 were observed with the loss of NEDD4-2 *(*N42^K^*)*, but not in the db/db mice alone at this age (Supplementary Fig. 4). Hence, further loss of NEDD4-2 from db/db mice does not exacerbate kidney pathology and presents similar to loss of NEDD4-2 alone. However, compared to WT, increased urinary albumin was observed in all lines, suggesting some loss of kidney function even at 12 weeks in the db/db mice (Fig. 5D). When normalised to creatinine, the urinary albumin:creatinine ratio (uACR) was increased in N42^K^ and N42^K^;db/db mice. GFR was significantly decreased in N42^K^;db/db mice, suggesting reduced kidney function. Hence, loss of NEDD4-2 causes similar kidney disease in WT non-diabetic and db/db diabetic mice.

### Correction of metabolic parameters in N42^K^;db/db mice

**Fig. 6:**
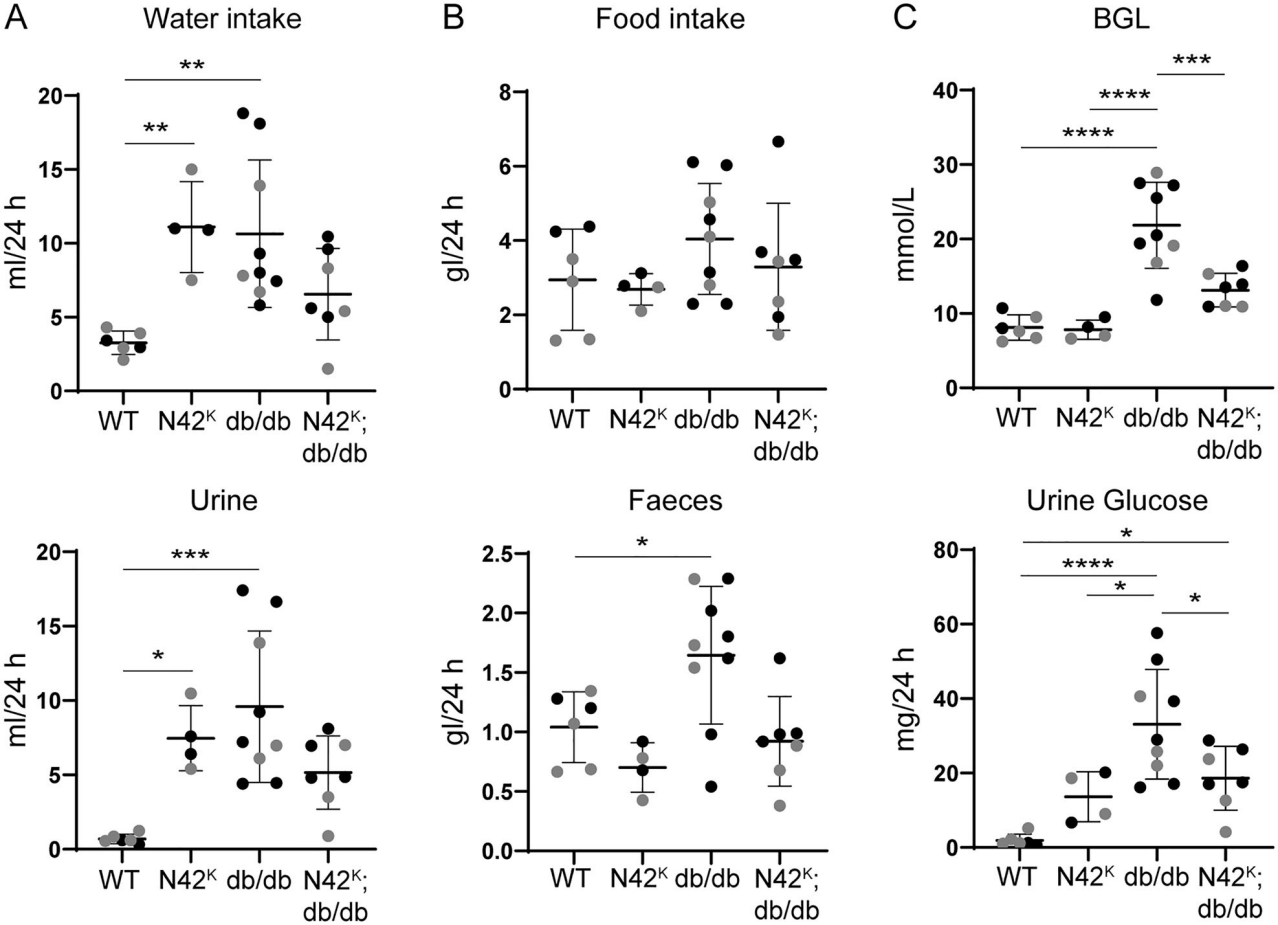
24 h metabolic parameters are corrected in N42^K^;db/db mice. **A**, **B** 12-week-old mice housed in metabolic cages with free access to food and water. **C** Fasting BGL measured prior to metabolic cage housing, urine glucose measured from 24 h urine samples. n = 4–9, data presented as mean ± SD and analysed by one-way ANOVA. *P < 0.05, **P < 0.01, ***P < 0.005, ****P < 0.001.

Diabetes is associated with increased thirst (polydipsia) and urination (polyuria) hence, metabolic cages were used to assess water and food intake. Similar to what we have previously shown [12], N42^K^ mice displayed an increase in water intake over 24 h, which correlated to increased urine output, due to their kidney pathology (Fig. 6A). This was also apparent in db/db mice, as expected [15]. However, interestingly, water intake and urination in N42^K^;db/db mice were similar to WT animals. No significant changes in food intake were observed, although the db/db mice produced more faeces (Fig. 6B). Analysis of 24 h urine collection revealed a decrease in osmolarity and creatinine in N42^K^, db/db and N42^K^;db/db lines (Table 1). Despite the increase in ENaC protein, we observed no significant changes in urinary Na^+^ excretion, however when normalised to creatinine, K^+^ was elevated in the urine of db/db mice and corrected by further loss of NEDD4-2, suggesting that NEDD4-2 contributes to electrolyte regulation (Table 1). Unlike in earlier studies [12], there were no significant changes in serum levels of Na^+^ and K^+^, perhaps due to background, the small sample size and variation in food and water intake.

**Table 1:** Urine and serum analysis of mice.

	WT	*N42*^*K*^	db/db	*N42*^*K*^*;db/db*
URINE				
Osmolarity (mOsmol/kg)	1920.33 ± 150.40 (3)	449.00 ± 181.44**** (4)	1280.00 ± 256.14* (5)	1166.00 ± 270.64** (5)
Creatinine (Cr) (mM)	3.15 ± 1.05 (4)	0.45 ± 0.26**** (4)	0.26 ± 0.13**** (5)	0.54 ± 0.31**** (5)
Na^+^/Cr (10^3^)	49.500 ± 19.50 (4)	113.25 ± 80.63 (4)	121.00 ± 24.99 (4)	63.00 ± 21.66 (5)
K^+^/Cr (10^3^)	96.85 ± 9.12 (4)	171.23 ± 16.78 (4)	291.40 ± 87.99** (4)	204.32 ± 23.36 (5)
SERUM				
Urea (mmol/L)	7.45 ± 1.07 (4)	16.90 ± 5.17** (4)	11.74 ± 2.64 (5)	10.08 ± 2.27 (4)
Creatinine (mmol/L)	11.00 ± 1.63 (4)	19.75 ± 3.20* (4)	5.75 ± 0.96 (4)	14.00 ± 7.39 (4)
Na^+^ (mM)	149.00 ± 4.08 (4)	155.00 ± 1.15 (4)	143.20 ± 6.91 (5)	151.80 ± 5.76 (5)
K^+^ (mM)	8.80 ± 3.98 (3)	8.30 ± 0.72 (4)	8.88 ± 0.43 (3)	9.36 ± 0.71 (3)
Ca^2+^ (mM)	2.58 ± 0.21 (4)	3.03 ± 0.19 (4)	2.59 ± 0.44 (5)	2.52 ± 0.59 (4)
HCO_3_^−^ (mM)	21.00 ± 3.92 (4)	26.67 ± 2.08 (3)	18.60 ± 4.67 (5)	18.50 ± 1.91 (4)
Glucose (mM)	16.95 ± 2.24 (4)	14.33 ± 2.59 (3)	38.68 ± 3.53**** (5)	21.30 ± 4.96 (4)
Globulin (g/L)	37.00 ± 1.83 (4)	41.67 ± 3.51 (3)	41.00 ± 3.81 (5)	44.25 ± 3.86* (4)
Albumin (g/L)	15.25 ± 2.06 (4)	17.00 ± 0.00 (3)	17.60 ± 1.67 (5)	17.75 ± 2.50 (4)
Protein (g/L)	52.25 ± 2.63 (4)	57.00 ± 4.40 (4)	58.60 ± 5.32 (5)	62.80 ± 5.63* (5)

Data presented as mean ± SD for number of mice (*n*) indicated in parentheses. Significance was determined against WT using a one-way ANOVA for non-normally distributed data.

*P < 0.05, **P < 0.01, ***P < 0.0005, ****P < 0.0001.

Fasting blood glucose levels (BGL) showed an expected increase in the db/db mice in male and female animals (Fig. 6C). Strikingly, this was corrected in the N42^K^;db/db mice, as glucose levels were similar to WT animals. Similarly, non-fasting serum levels of glucose were also elevated only in the db/db mice (Table 1). High glucose levels also resulted in glucosuria in db/db mice, which was significantly decreased in N42^K^;db/db but still higher than in WT mice (Fig. 6C), suggesting that the lower BGL in these mice is not due a defect in glucose reabsorption alone.

### Restoration of elevated aldosterone and insulin signalling in N42^K^;db/db mice

**Fig. 7:**
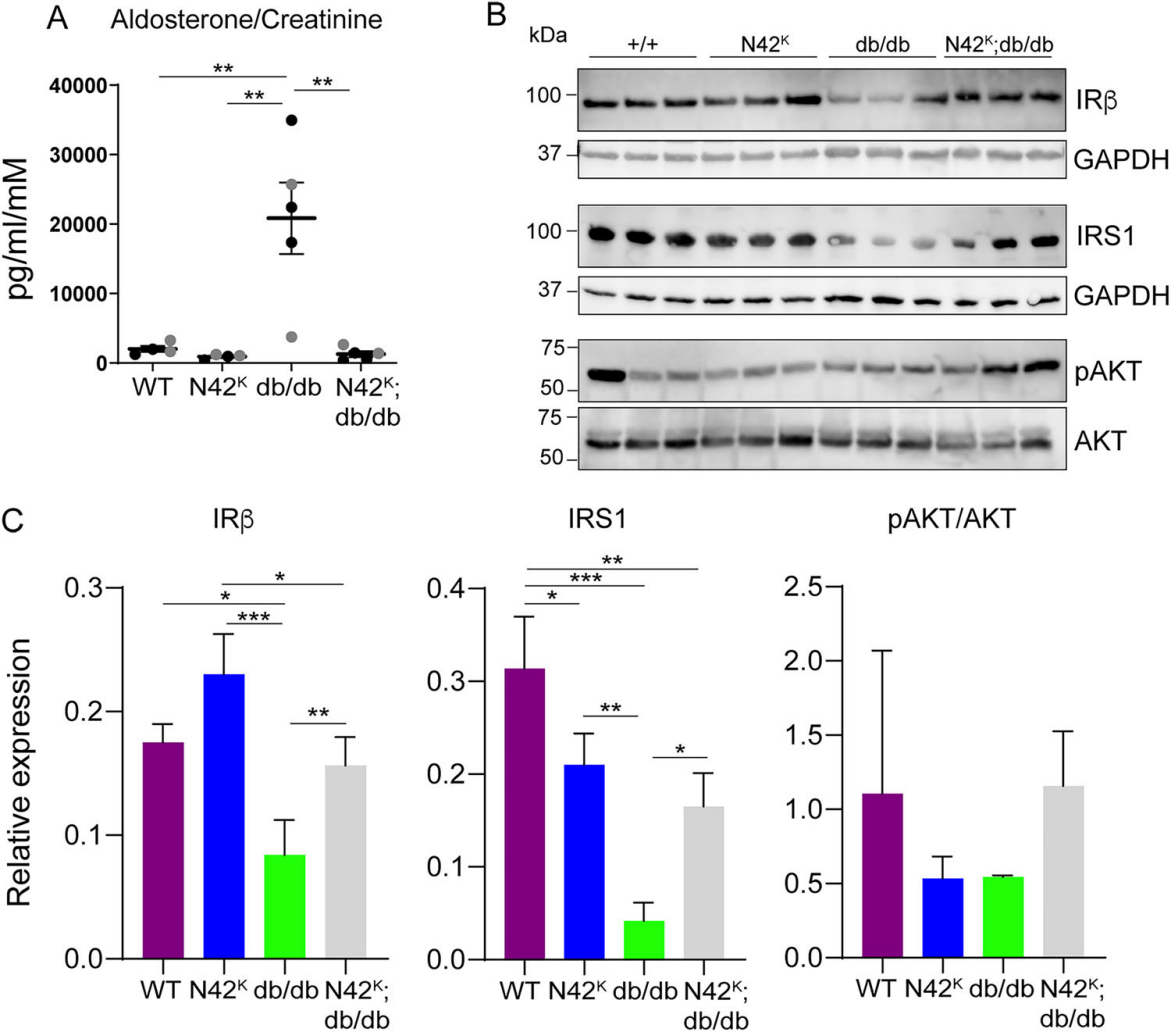
Aldosterone and insulin signalling is restored in N42^K^;db/db mice. **A** Urine aldosterone levels normalised to creatinine, n = 4–5. **B** Liver lysates assessed for insulin receptor beta (IRβ), insulin receptor substrate-1 (IRS-1) and pAKT473/AKT. **C** Quantitation of IRβ and IRS-1 to GAPDH, ratio of pAKT/AKT, n = 3. Data presented as mean ± SEM for (A) or mean ± SD for (**C**) and analysed by one-way ANOVA. *P < 0.05, **P < 0.01, ***P < 0.005.

Hyperglycaemia in db/db mice is caused by mutation in the leptin receptor, which affects hypothalamic responses and leads to obesity and insulin resistance [15]. Additionally, elevated plasma aldosterone levels, which is a feature of patients with diabetes [17], is also evident in db/db mice and contributes to elevated BGL [18, 19]. Aldosterone secretion is regulated by serum K^+^ levels and the renin-angiotensin system [20]. Given that we have previously observed reduced aldosterone levels in kidney-specific *Nedd4-2* knockout mice (N42^K^) due to increased Na^+^/reduced K^+^ retention [12, 14], we next assessed whether the reduction of *Nedd4-2* in db/db mice (N42^K^;db/db) affected aldosterone levels. When normalised to creatinine, urine aldosterone levels were significantly elevated in db/db mice, and this was corrected in the N42^K^;db/db mice (Fig. 7a). There was a similar trend in blood aldosterone levels, although this was not significant due to low sample numbers (Supplementary Fig. 5). High aldosterone levels in diabetes has been shown to contribute to insulin resistance, in part due to resulting hypokalaemia [18], leading to elevated BGL. Hence, we assessed whether loss of *Nedd4-2* in db/db mice may restore insulin sensitivity. There was an increase in insulin-positive islet area in db/db mice, but this was similar with additional loss of NEDD4-2, suggesting that changes in insulin secretion do not explain correction of BGL (Supplementary Fig. 6). However, evidence of altered insulin signalling was observed in the liver, a primary tissue where insulin resistance occurs. As demonstrated previously [21], levels of the insulin receptor (IRβ) were reduced in db/db mice, and this was restored to WT levels by the loss of NEDD4-2 (Fig. 7B, C). Furthermore, we observed decreased levels of IRS-1 in db/db livers, which is associated with aldosterone-induced insulin resistance [22, 23]. This was restored with renal loss of NEDD4-2. Downstream insulin signalling was also partially restored, as evidenced by a trend towards increased pAKT in the N42^K^;db/db mice (Fig. 7B, C). Based on these data, we conclude that the loss of NEDD4-2 from diabetic mice corrects aldosterone levels and restores insulin signalling in db/db mice to improve BGL.

## Discussion

The current study reports a reduction of *Nedd4-2*, and an increase in its substrates in the kidneys of a mouse model of type 2 diabetes (db/db) that progresses with the development of disease. Further genetic ablation of *Nedd4-2* from the tubules of db/db mice resulted in kidney pathology similar to knockout of *Nedd4-2* in a WT background, without exacerbating DKD progression. However, it corrected metabolic parameters and aldosterone levels, and normalised BGL in mice of both sexes, suggesting that inhibition of *Nedd4-2* could be protective in glucose control in diabetes.

Down-regulation of *NEDD4L* mRNA has been reported previously in patients with DKD, displaying elevated A1c (glycated haemoglobin), pathological changes and albuminuria [9]. Here we demonstrate a similar finding in the db/db mouse model of type 2 diabetes, leading to a decrease in the protein levels of NEDD4-2 and subsequent increase in its substrates. Interestingly, the changes in *Nedd4-2* levels in this study were apparent from 4 weeks of age in male mice, before any significant increases in BGL or body weight, suggesting that this is an early event in disease and not a result of pathological changes in the kidney. A significant down-regulation of *Nedd4-2* in female mice was not apparent until 12 weeks of age, raising the possibility that the earlier down-regulation of *Nedd4-2* in males may correlate with the increased risk of male patients developing kidney disease [24].

The effects on ion secretion seen in this study are similar to those seen in patients with DKD [9], likely due to elevation of NEDD4-2 substrates. Increased levels of ENaC subunits and NCC have also been observed in the mouse model of Type 1 diabetes induced by streptozotocin (STZ) [25] and in Type 2 diabetes, partly attributed to increased insulin [11]. Insulin leads to stimulation of the aldosterone and glucocorticoid-responsive gene, SGK1, which in turn increases levels of ENaC and NCC at the membrane by inhibiting the action of NEDD4-2 [26, 27]. ENaC and SGK1 can be upregulated by high glucose [28], and results from this study suggest that this may be through the action of NEDD4-2.

Our previous work demonstrated that loss of *Nedd4-2* from kidney tubules leads to kidney disease caused at least in part by high ENaC activity [12]. Given that db/db mice develop DKD from 12-16 weeks, we hypothesised that reduced NEDD4-2 may contribute to kidney injury characteristics in DKD and that further genetic loss of *Nedd4-2* from these mice may lead to earlier onset or more severe disease. At 12 weeks of age, when kidney function is initially compromised in both N42^K^ and db/db mice (as shown by presence of urinary albumin), pathology in N42^K^;db/db mice is indistinguishable from N42^K^ mice alone, with minimal changes in kidney function. Surprisingly however, this cross resulted in a correction of BGL and metabolic parameters, that are normally aberrant in these individual strains [14, 29]. The correction of BGL in N42^K^;db/db mice, together with a reduction of urinary glucose excretion suggests that the loss of NEDD4-2 from the tubules of these mice, despite causing significant kidney pathology, restores glucose homeostasis, and leads to a reduction in drinking and urine output.

Several possibilities exist as to how loss of renal NEDD4-2 is exerting this systemic effect, likely involving elevated expression of its substrates and subsequent alterations in ion secretion and reabsorption. In particular, we have previously demonstrated that elevated ENaC levels and resulting increased sodium reabsorption lead to a decrease in aldosterone levels in N42^K^ mice [12, 14]. In diabetes, aldosterone levels are elevated [18, 30], both in patients [17] and in the db/db mouse model [31], as this hormone increases the expression of ENaC to enhance sodium reabsorption [32]. Importantly, we observed correction of elevated aldosterone levels in N42^K^;db/db mice, together with restored secretion of K^+^, perhaps due to modest but non-significant changes in serum levels of Na^+^ and K^+^.

Elevated aldosterone in diabetes has been shown to contribute to increased BGL [30]. Hence, the reduction of aldosterone levels that we observe in diabetic mice caused by loss of NEDD4-2 may explain, at least in part, the correction of BGL in these animals. Mechanistically, elevated aldosterone contributes to insulin resistance in several tissues, including adipose, liver and muscle [18], contributing to impaired cellular glucose uptake and utilisation, via several mechanisms, including hypokalameia [33]. Indeed, we observed a decrease in IRβ and its substrate IRS-1 in the db/db mice, associated with insulin resistance [18], that was restored by further loss of NEDD4-2. Together with elevation of pAKT, this suggests that decreased aldosterone and increased insulin signalling in the N42^K^;db/db mice contribute to lower BGL.

**Fig. 8:**
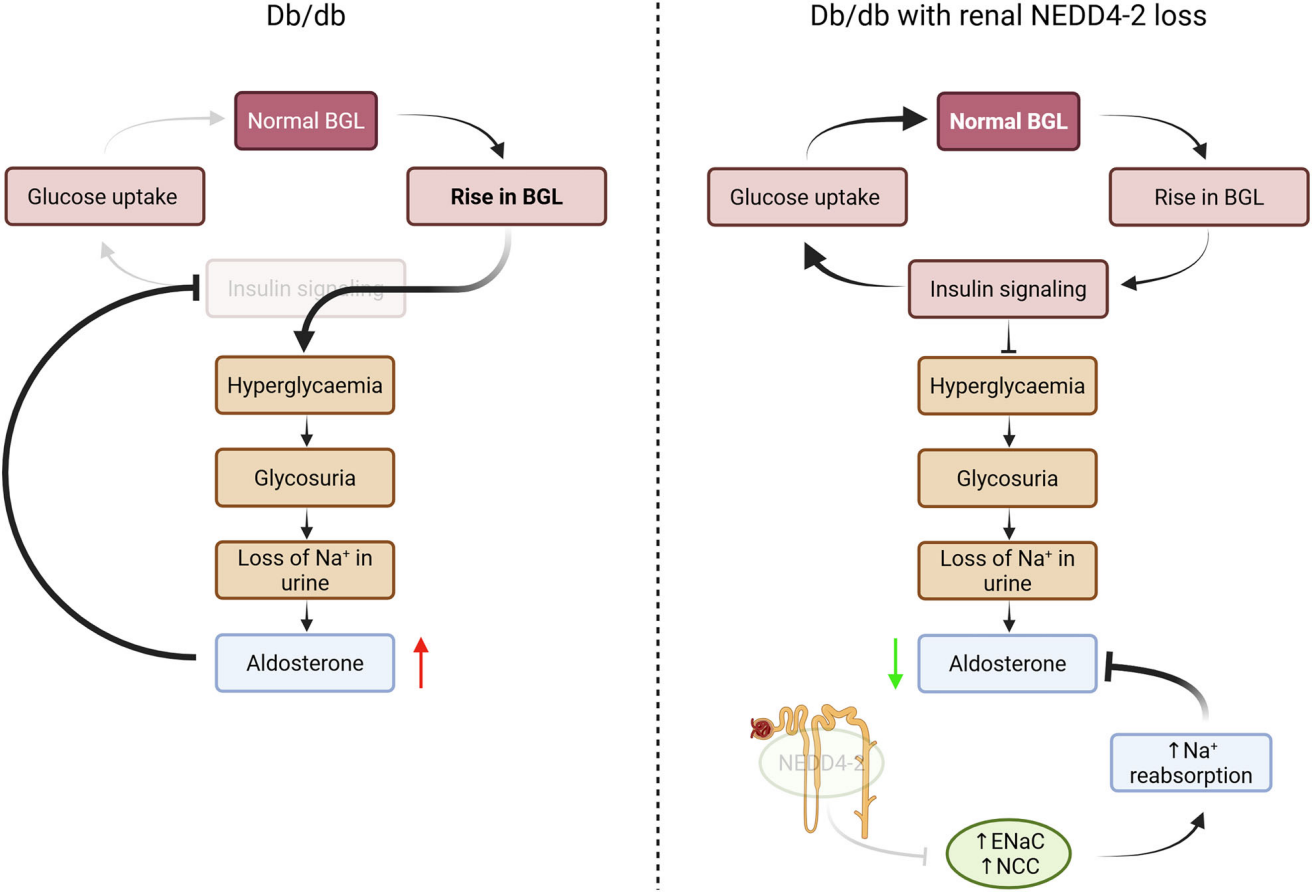
Model of renal NEDD4-2 loss in db/db. In diabetic db/db mice, lack of insulin signalling results in hyperglycaemia, subsequent glycosuria, loss of urinary Na^+^ (increased Na^+^ excretion) and increased aldosterone levels. Elevated aldosterone can further inhibit insulin signalling. Loss of renal NEDD4-2 results in elevated levels of ENaC and NCC (reported previously, and in this study) to increase Na^+^ reabsorption. High Na^+^ can inhibit aldosterone levels to restore insulin signalling, allowing cellular glucose uptake and restoring BGL to normal levels.

In conclusion, our study demonstrates that reduction of *Nedd4-2* is an early event in the db/db mouse, driving elevated expression of ENaC and NCC and alterations in electrolyte balance. Further loss of renal *Nedd4-2* has paradoxical effects; it causes mild kidney disease, but is also able to rescue BGL in db/db mice, through elevation of ENaC and reduction of aldosterone (Fig. 8). Clinically, the reduction of aldosterone signalling via mineralocorticoid receptor antagonists has been used to treat DKD, although it comes with significant limitations [34]. More recently, inhibition of aldosterone synthase has been shown to lower BGL in rats [35] and provide protection against chronic kidney disease [36], and has promise for treating diabetic complications [37]. The finding that NEDD4L reduction and subsequent ENaC elevation in diabetes reduces aldosterone levels and therefore BGL provides mechanistic insight into how reduction of aldosterone may improve glucose homeostasis. Furthermore, our data suggests that NEDD4L expression might affect aldosterone levels in diabetic patients and, therefore treatment responses to aldosterone inhibition. Hence, further work may reveal the importance of this gene in the treatment of hyperglycaemia in patients.

## Materials and methods

### Animal models

All animal studies were approved by the UniSA Ethics Committee (see Ethics Statement). Kidney-specific *Nedd4-2*-deficient mice (Nedd4-2Ksp1.3, N42^K^) were generated in our laboratory previously [12]. db/db (B6.BKS(D)-*Lepr*^*db*^/J) mice were kindly provided by A/Prof Ross Laybutt (Garvan Institute, Sydney, Australia) and were originally from JAX Laboratories. Both strains were bred at the University of South Australia's core animal facility (Adelaide, Australia) under specific pathogen-free conditions, and crossed together to generate the N42^K^;db/db line, backcrossed to the B6.BKS(D)-*Lepr*^*db*^/J strain for at least 10 generations before use, with littermates used as controls to minimise changes due to genetic background [38]. Groups were assigned based on genotypes and not blinded. For the db/db study, mice were fasted for 6 h, blood glucose measured by tail prick and glucometer, followed by CO_2_ asphyxiation before dissection of organs. For the N42^K^;db/db study, mice were fasted for 6 h and blood glucose measured. Mice were housed in metabolic cages for a 6 h training session, then a 24 h session to record parameters the following day. Mice were provided *ad libitum* access to deionized drinking water and pelleted chow. At the end of the experiment, urine was collected, mice were anaesthetised and blood collected by cardiac puncture. Organs (kidney, pancreas and liver) were dissected after cervical dislocation. Capsules were removed from the kidneys and one placed into Histochoice reagent (ProSciTech, Kirwan, QLD, Australia) for histological analysis of paraffin-embedded or frozen samples. For paraffin samples, organs were transferred to 70% ethanol and then embedded in paraffin. Organs for frozen sectioning were soaked in 30% sucrose overnight before being embedded in OCT (ProSciTech, Kirwan, QLD, Australia). A portion of tissue was snap frozen in liquid nitrogen for protein or mRNA analysis.

### Histological analysis

Kidney sections (5 μm) were cut using a paraffin microtome, de-paraffinized with xylene and dehydrated through a graded series of ethanol. Slides were stained with Hematoxylin and Eosin using standard protocols. Digital images were acquired by using a NanoZoomer (Hamamatsu). Kidney scoring was conducted blinded as we have previously described [39], by two independent investigators.

### Quantitative real-time PCR

Total RNA was isolated from half of each kidney using TRIzol Reagent (Life Technologies) and RNA was reverse-transcribed with a high-capacity cDNA reverse transcription kit (Applied Biosciences). qRT-PCR was performed and analyzed as described [40], where all data were normalised to TBP (TATA box binding protein) levels. Primer sequences are Nedd4-2 (N42) F: GCAGAAGGACAGAGGGTCG, R: ACGGGATTCTCCCTCCTCTT. Collagen-1 (Col1a1) F: CGGAGAAGAAGGAAAACGAGGAG, R: CACCATCAGCACCAGGGAAAC. Vimentin F: CGGCTGCGAGAGAAATTGC, R: CCACTTTCCGTTCAAGGTCAAG. Kidney injury molecule 1 (Kim-1) F: TGGTTGCCTTCCGTGTCTCT, R: TCAGCTCGGGAATGCACAA, TGF-β1 F: GATACGCCTGAGTGGCTGTC, R: AAGCCCTGTATTCCGTCTCC. Il1β F: CCAGAGATACAAAGAAATGATGG, R: ACTCCAGAAGACCAGAGGAAAT. NCC F: ACAGTGAGAAGAGCCCTGGA, R: GATGATGAGCCAAGTCAGCA. TATA-box binding protein (TBP), F: CAAACCCAGAATTGTTCTCCTT, R: ATGTGGTCTTCCTGAATCCCT.

### Immunoblotting

Kidney or liver samples were lysed in ice-cold extraction buffer at pH 7.5 (50 mM Tris-HCl pH 7.5, 1 mM EDTA, 1 mM EGTA, 0.27 M sucrose, 0.1% β-mercaptoethanol and HALT protease and phosphatase inhibitor cocktail [Thermo Fisher Scientific]). Tissue was homogenised, frozen in liquid nitrogen, immediately thawed, and incubated at 4 °C on a nutator for 30 min and centrifuged at 13,000 rpm for 5 min. Supernatant protein (25 μg) was combined with protein load buffer (100 mM Tris-HCl pH 6.8, 200 mM DTT, 4% SDS, 0.2% bromophenol blue, 20% glycerol), heated at 100 °C for 5 min (or 37 °C for 30 min for SGLTs), loaded onto 4–20% precast SDS-PAGE gels (Bio-Rad) and transferred to PVDF membrane using the Trans-blot Turbo instrument (Bio-Rad). Membranes were blocked with 5% BSA in TBS-T (Tris-buffered saline, 0.05% Tween 20) and primary antibodies added; anti-NEDD4-2 (in house [41]), anti-β-actin (Sigma, AM4302), anti-GAPDH (CST, 14C10), anti-α-ENaC and anti-γ-ENaC [42]; rabbit anti-β-ENaC [43], rabbit anti-NCC (Abcam, ab3553), anti-IRβ (CST, 23413), anti-IRS1 (Abclonal, A0245), anti-pAKT473 (CST, D9E) or anti-panAKT (CST, 40D4). HRP secondary antibodies (Millipore) were added at 1:2000 and developed with ECL Prime (GE Healthcare) or West Femto (Thermo Scientific). β-actin and GAPDH were developed using Cy5 secondary antibody (GE Healthcare). Images were acquired on a ChemiDoc Touch Imager (BioRad). Quantitation was conducted using Image Lab Software (BioRad), with each band normalised to β-actin or GAPDH.

### Immunostaining

Paraffin sections (kidney or pancreas, 5 μm) were deparaffinized and hydrated in a graded ethanol series. Heat-mediated antigen retrieval was carried out by boiling for 10 min in 10 mM citric acid solution (pH 6). Tissue sections were blocked with 10% goat serum. Primary antibodies used were as above: Nedd4-2, α, β and γ ENaC, NCC or anti-insulin (Abcam, ab195956) and rhodamine-labelled DBA (Vector Laboratories, RL-1032). Sections were incubated with primary antibody overnight at 4 °C and with secondary for 3 h at room temperature (fluorescently tagged AlexaFluor-488 or AlexaFluor-568, Thermo Fisher Scientific), counterstained with Hoechst 33342 (Thermo Fisher) and mounted in Prolong Gold Antifade reagent (Invitrogen). Stained samples were imaged using an LSM 800 confocal microscope using Zen 2011 (Black Edition) version 8.1.5.484 (Carl Zeiss Microscopy). Fluorescence intensity or islet size was calculated using ImageJ, with intensity normalised to area size of tubule for Nedd4-2 staining.

### Blood and urine analyses

24 h urine samples were measured for glucose concentration using a Glucose Colorimetric/Fluorometric Assay Kit (BioVision, K606-100), or albumin using a Mouse Albumin ELISA Kit (Abcam, ab108792), using 1 μl urine as per manufacturer’s instructions. Aldosterone was measured in urine and serum using an Aldosterone ELISA Kit (Abcam, ab136933) at a 1:20 dilution, as per manufacturer’s instructions, and normalised to creatinine levels for urine results. Electrolytes and other parameters of kidney function in blood (as in Table 1) were carried out by SA Pathology (Adelaide, Australia). Urine osmolality was measured using an Advanced 3320 osmometer (Advanced Instruments). Creatinine, Na^+^, K^+^ and Cl^−^ were measured using an Advia 2400 chemistry system (Siemens). GFR was calculated from blood and urine results as we have done previously [12].

### Statistical analysis of data

Statistical analysis was performed using GraphPad Prism software (v6.0). A Mann-Whitney test for non-parametric data was used to assess changes in blood/urine parameters in Table 1, and one-way ANOVA used to assess repeated measurements. Remaining data were analysed using unpaired 2-tailed Student’s t-test. A P value of ≤0.05 was considered significant. All values are presented as mean ± SEM (or ±SD for immunoblot quantitation), as indicated in the figure legends.

## Supplementary information

Supplementary Information

Original western blots

## Data Availability

All data generated from this study are presented in the main manuscript and supplementary information. Raw data are available on request.
