# Supplementary material for: Nedd4-2 ablation in kidney improves glycaemic control in diabetic mice
Source: Cell Death Dis. 2025 Jul 5;16(1):496. doi: 10.1038/s41419-025-07826-3 (PMC12228766; doi:10.1038/s41419-025-07826-3)
Supplement: Supplementary file 1 — Supplementary Information [file 41419_2025_7826_MOESM1_ESM.pdf]

Supplementary Information

Includes the following figures:

Supp. Fig. 1. Blood glucose levels (BGL) in db/db mice

Supp. Fig. 2. Whole body weights in db/db mice

Supp. Fig. 3. Immature ENaC quantification

Supp. Fig. 4. Markers of kidney damage

Supp. Fig. 5. Serum aldosterone levels

Supp. Fig. 6. Pancreatic insulin secretion

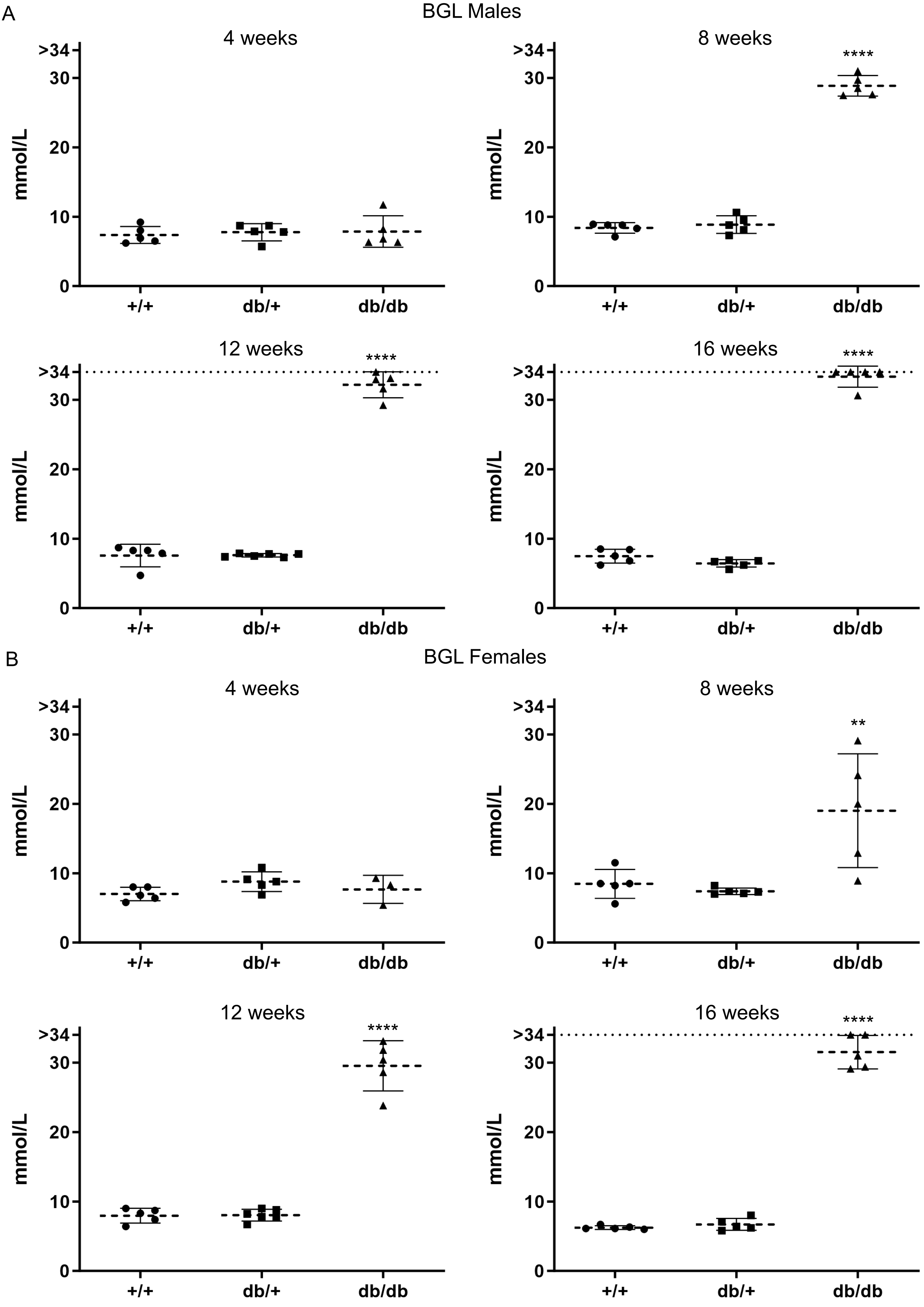

Supp. Fig. 1. Blood glucose levels (BGL) in mice. Fasting BGL was measured from tail prick at 4, 8, 12 or 16 weeks in males (A) and females (B). Dotted line indicated readings above the cut off range of 34 mmol/L. Data presented as mean ± SD and analysed by one-way ANOVA. **P < 0.01, ****P < 0.001.

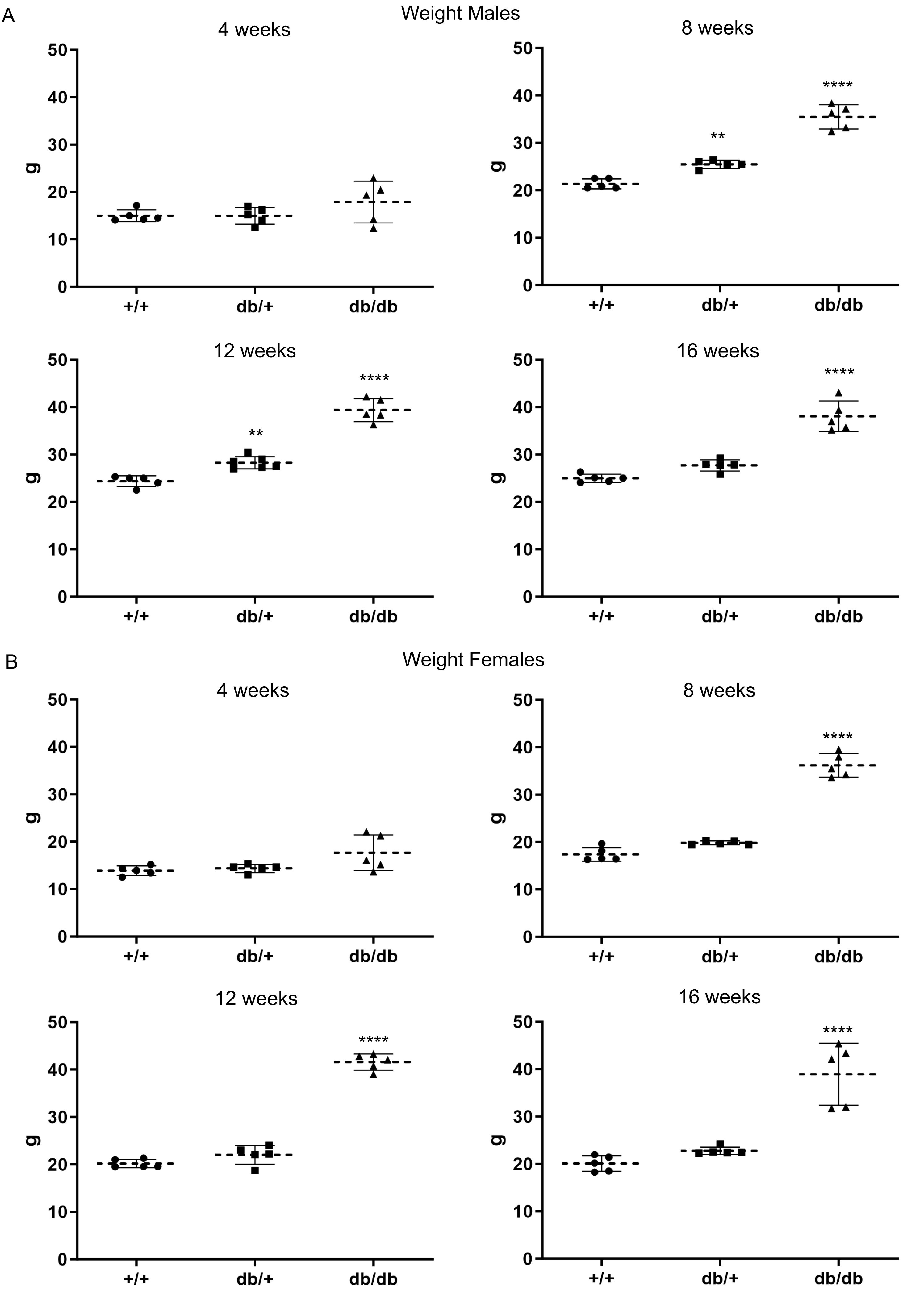

Supp. Fig. 2. Whole body weights in mice. At 4, 8, 12 or 16 weeks in males (A) and females (B). Data presented as mean ± SD and analysed by one-way ANOVA. ****$P < 0.001$.

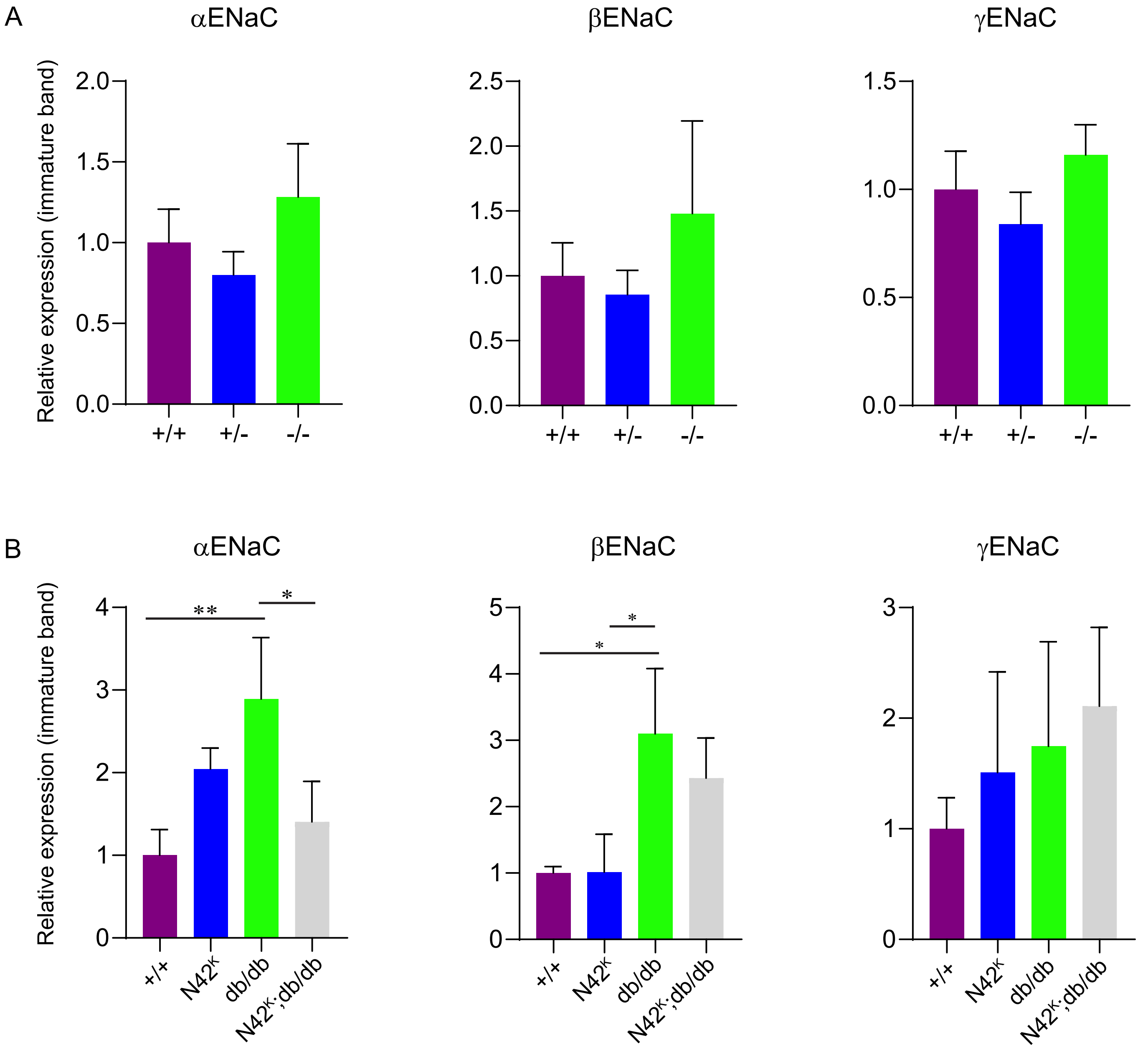

Supp. Fig. 3. ENaC quantitation. Immature ENaC band quantitated relative to β-actin from (A) main Figure 3 and (B) main Figure 4, n = 3 mice. αEnaC (95-110kDa), βENaC (96 kDa), γENaC (93 kDa). Data presented as mean ± SD and analysed by one-way ANOVA. *P < 0.05, **P < 0.01.

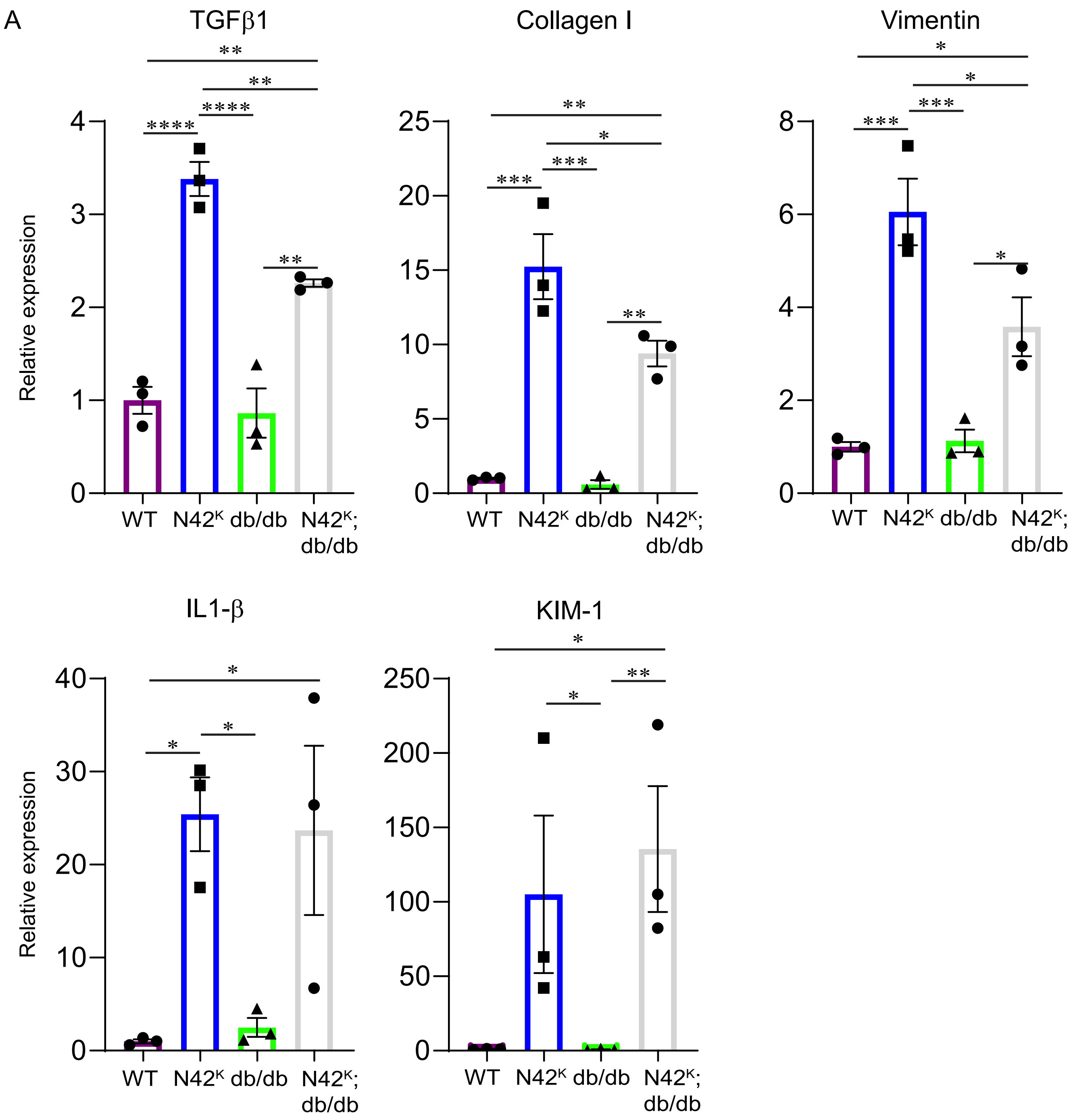

Supp. Fig. 4. Markers of kidney damage. Kidneys were assessed by qPCR for various markers of damage. Data presented as mean ± SEM and analysed by one-way ANOVA. *P < 0.05, **P < 0.01, ***P < 0.005, ****P < 0.001.

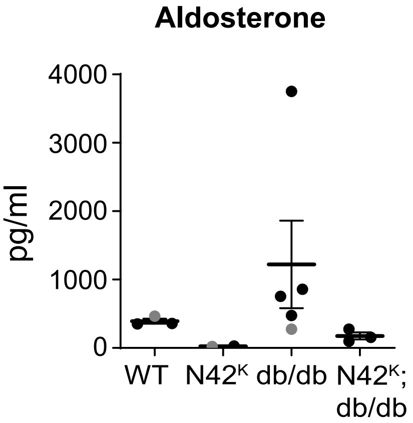

Supp. Fig. 5. Serum aldosterone levels. Serum assessed for aldosterone levels by ELISA. Data presented as mean ± SD and analysed by one-way ANOVA, no significance found.

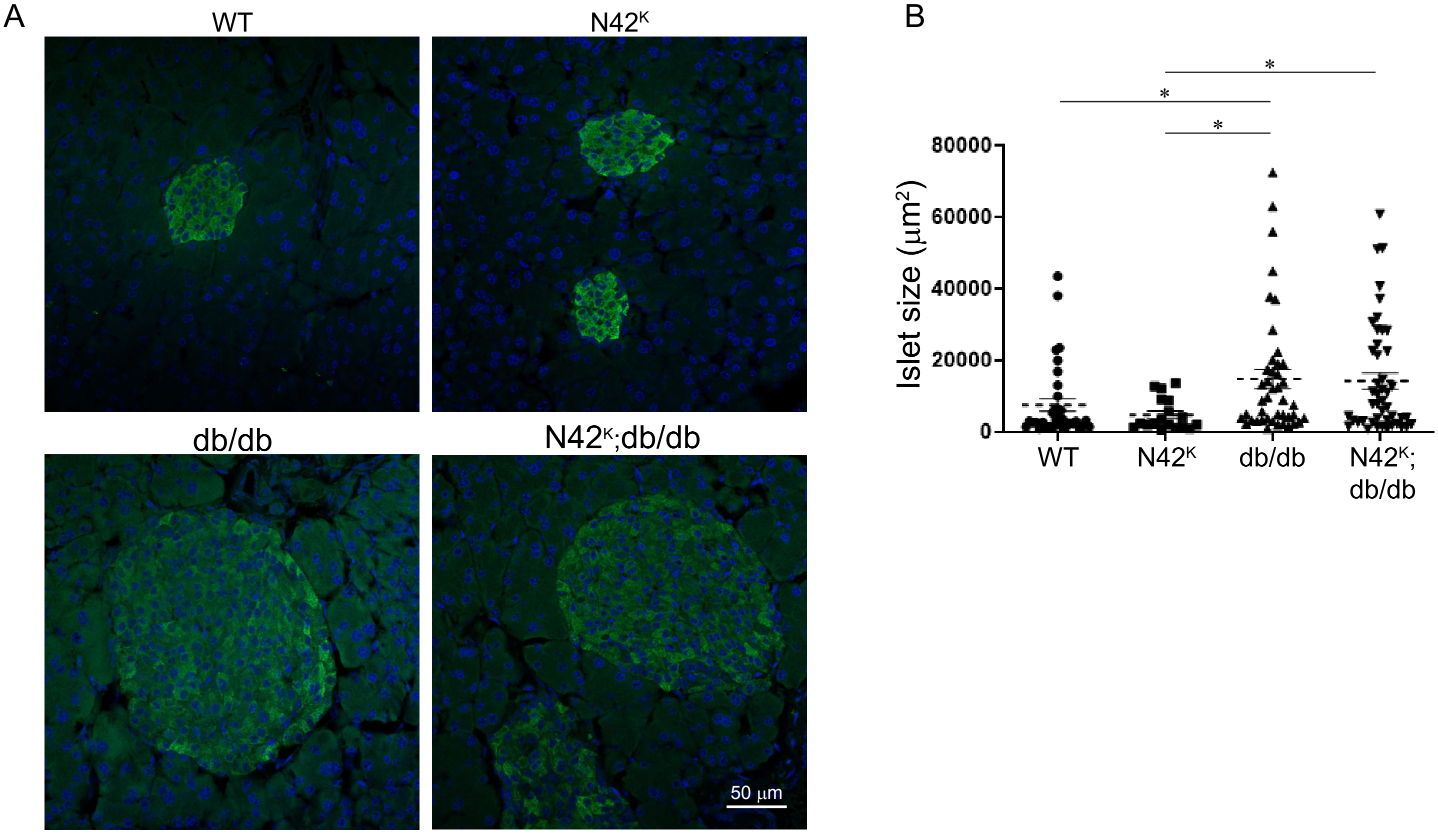

Supp. Fig. 6. Pancreatic insulin secretion . (A) Immunofluorescence of insulin-stained islets (green) with DNA (blue). (B) Insulin positive islet size measured, and data presented as mean ± SEM and analysed by one-way ANOVA. *P < 0.05.
