# Supplementary material for: Nedd4-2 ablation in kidney improves glycaemic control in diabetic mice
Source: Cell Death Dis. 2025 Jul 5;16(1):496. doi: 10.1038/s41419-025-07826-3 (PMC12228766; doi:10.1038/s41419-025-07826-3)

Figure 1C: Males

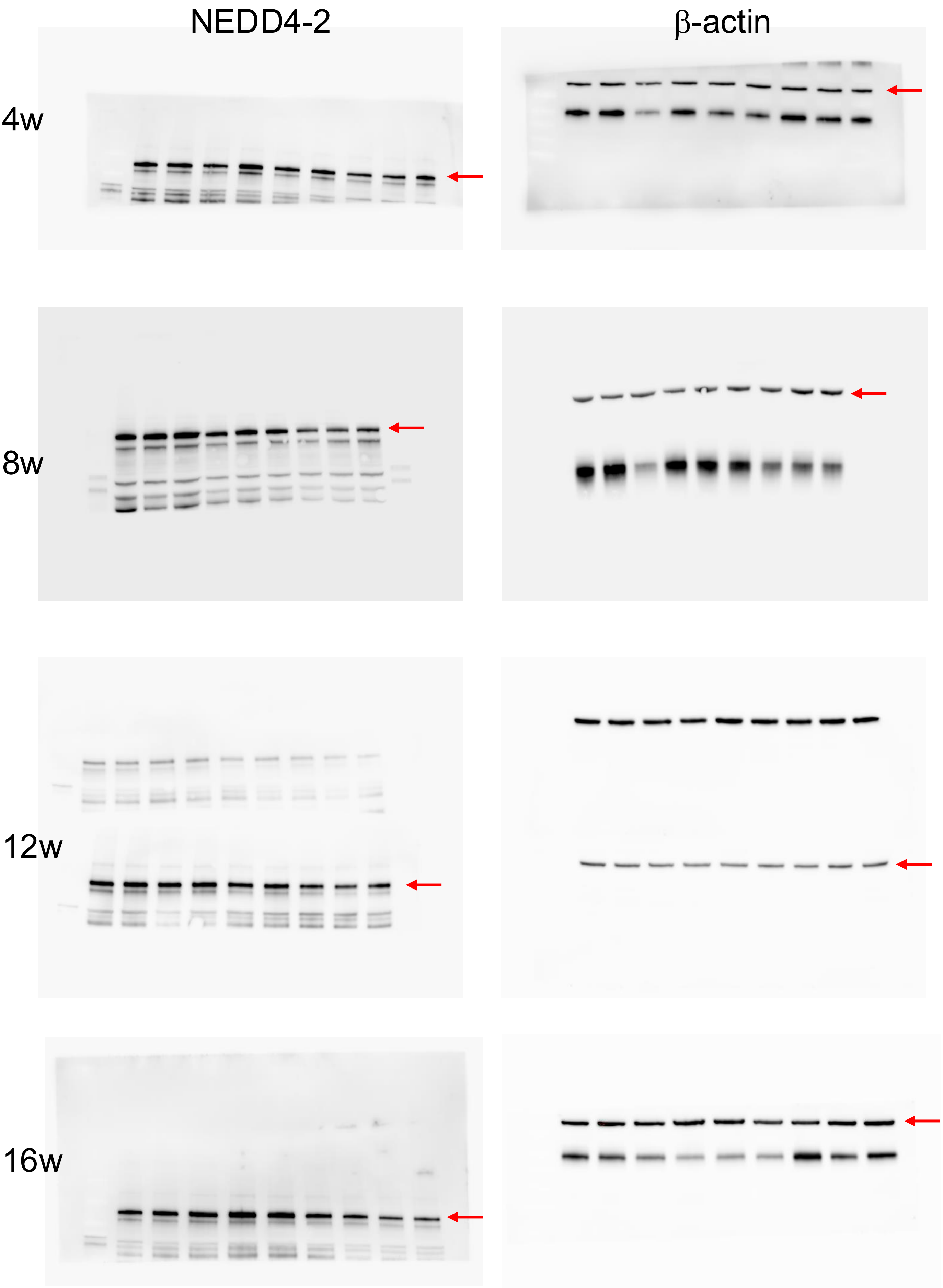

Figure 1C: Females

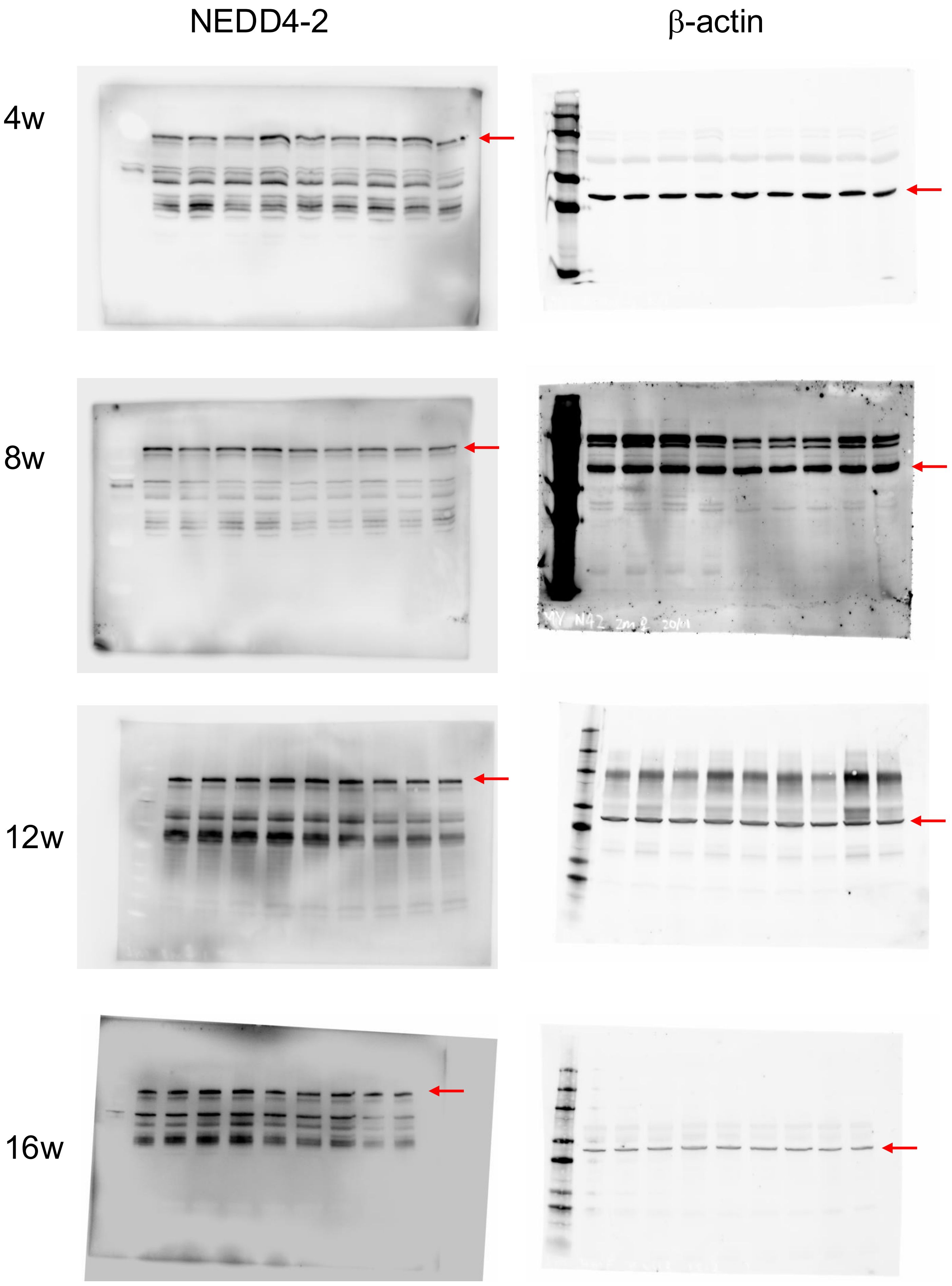

Figure 3B

αENaC

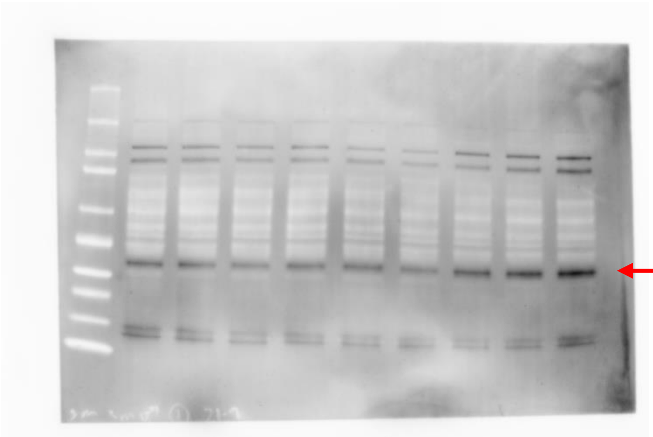

β-actin

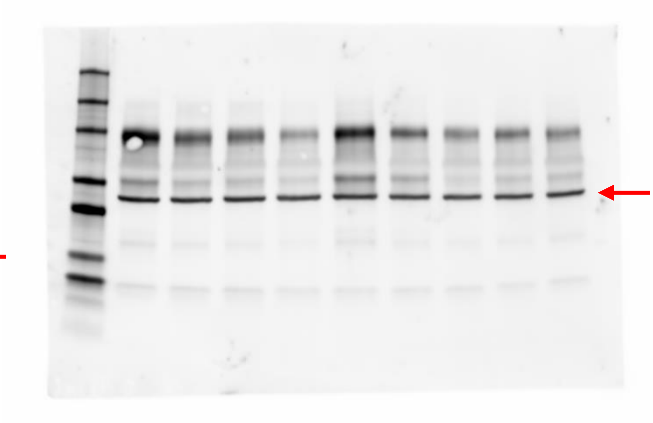

βENaC

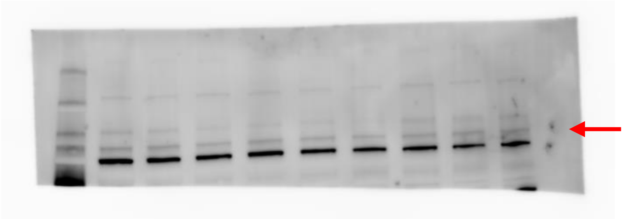

β-actin

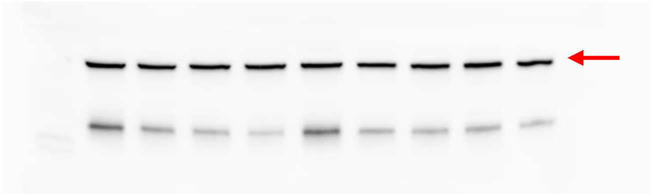

γENaC

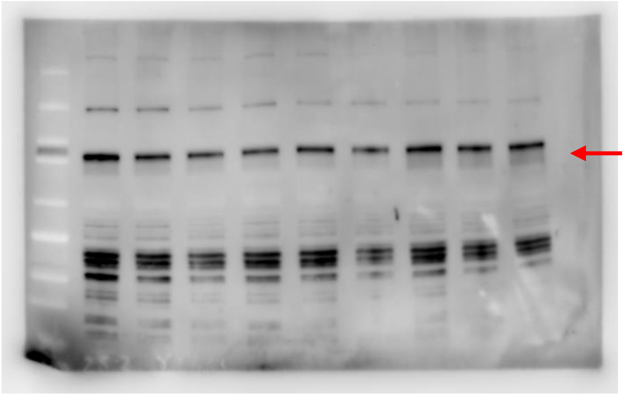

β-actin

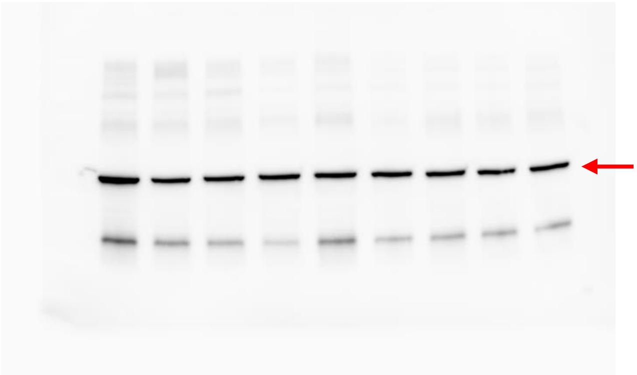

Figure 3E

NCC

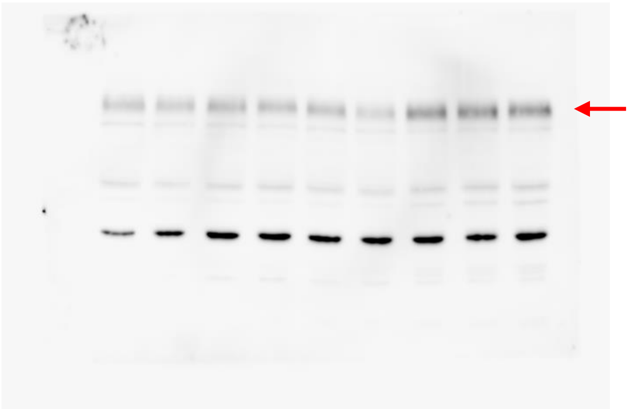

β-actin

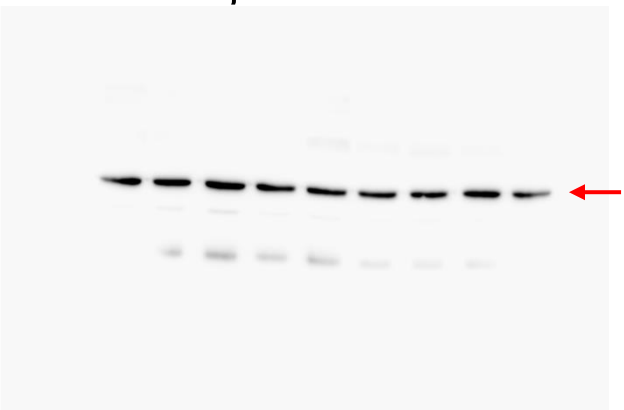

Figure 4B

NEDD4-2

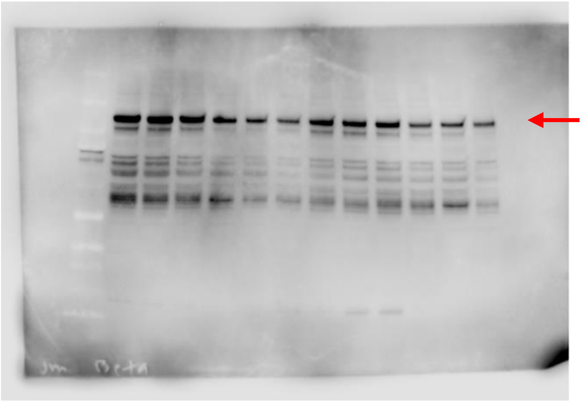

β-actin

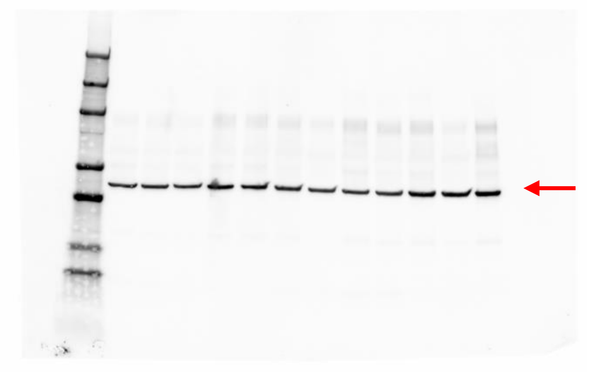

Figure 4D

αENaC

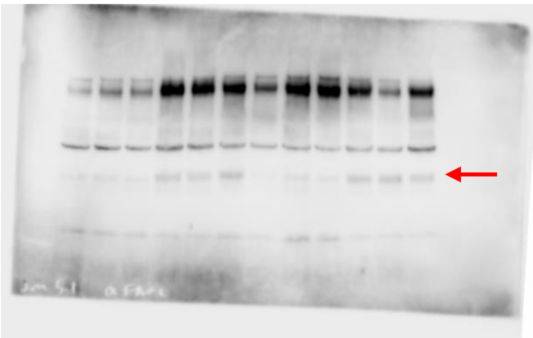

β-actin

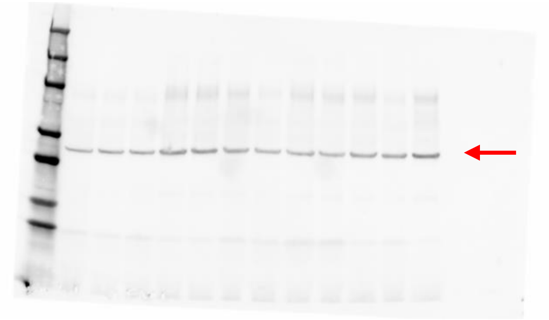

βENaC

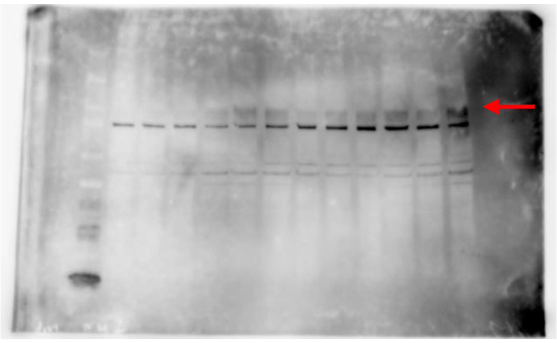

β-actin

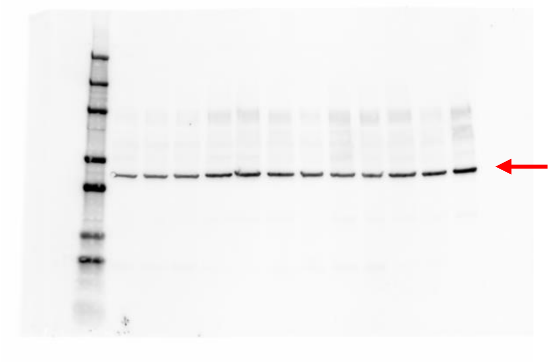

γENaC

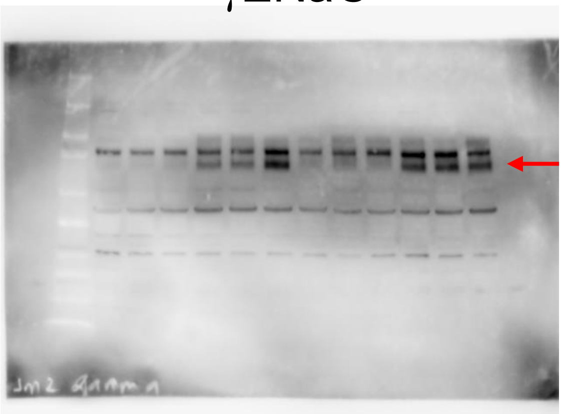

β-actin

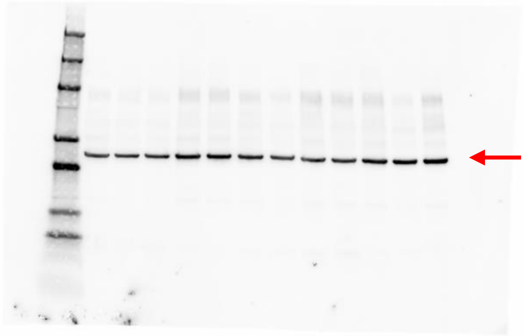

NCC

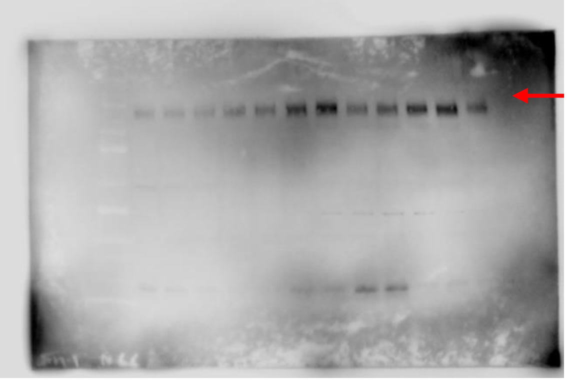

β-actin

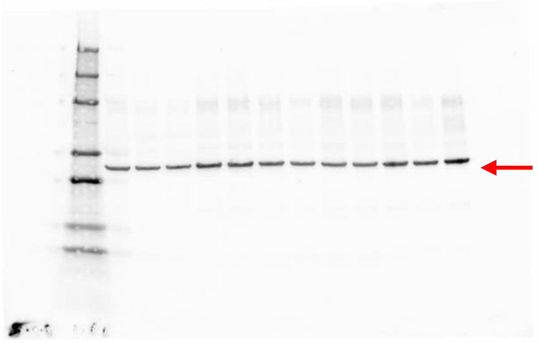

Figure 7B

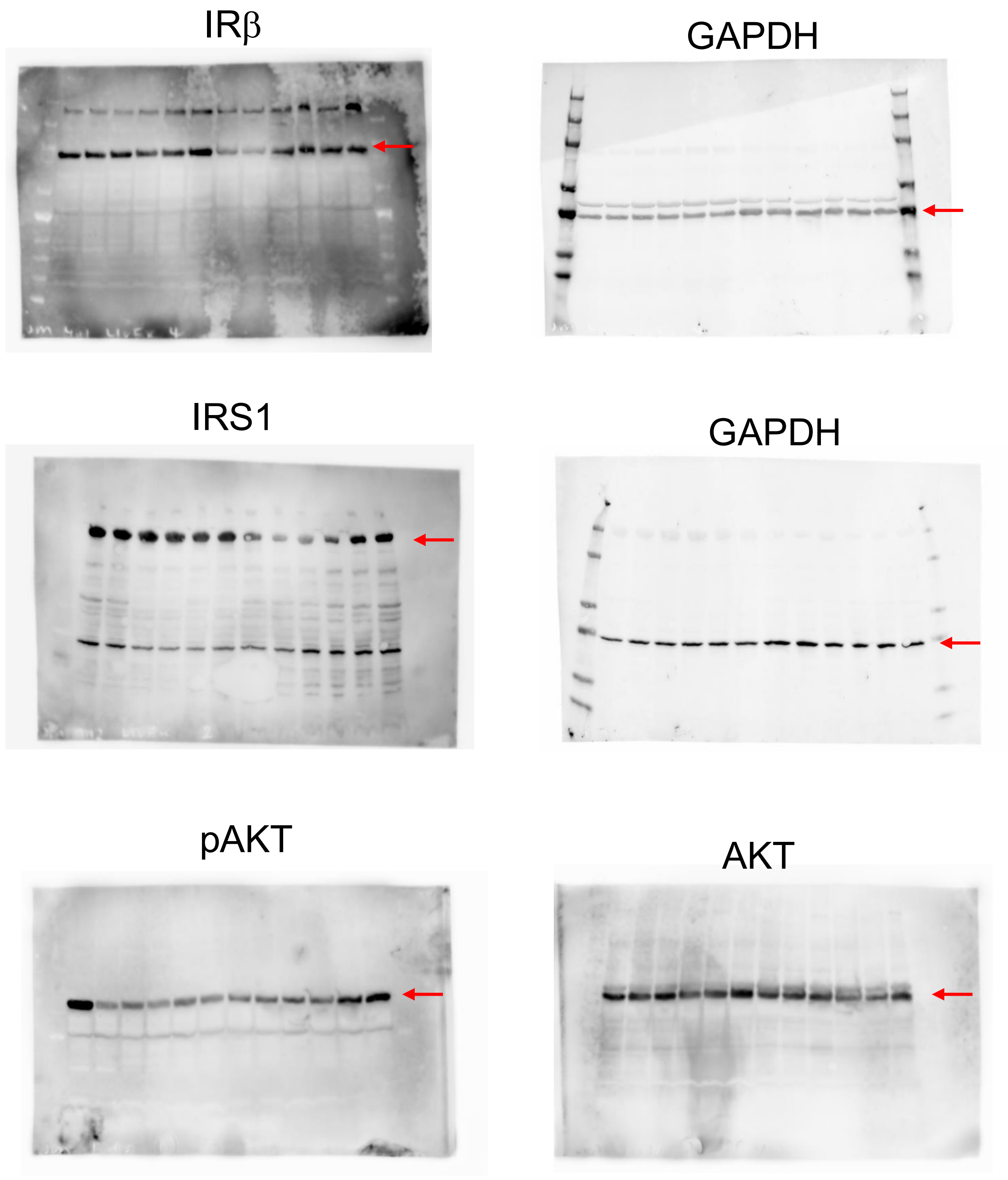

Supplement: Supplementary file 2 — Original western blots [file 41419_2025_7826_MOESM2_ESM.pdf]
